# Chemokine receptor CXCR7 activates Aurora Kinase A and promotes neuroendocrine prostate cancer growth

**DOI:** 10.1172/JCI166248

**Published:** 2023-08-01

**Authors:** Galina Gritsina, Ka-wing Fong, Xiaodong Lu, Zhuoyuan Lin, Wanqing Xie, Shivani Agarwal, Dong Lin, Gary E. Schiltz, Himisha Beltran, Eva Corey, Colm Morrissey, Yuzhuo Wang, Jonathan C. Zhao, Maha Hussain, Jindan Yu

**Affiliations:** 1 Division of Hematology/Oncology, Department of Medicine, Northwestern University Feinberg School of Medicine, Chicago, Illinois, USA.; 2 Department of Toxicology and Cancer Biology, University of Kentucky, Lexington, Kentucky, USA.; 3 Department of Urology, Emory University School of Medicine, Atlanta, Georgia, USA.; 4 Department of Urology, the Second Affiliated Hospital of Guangzhou Medical University, Guangzhou, China.; 5 Department of Experimental Therapeutics, BC Cancer Agency, Vancouver, British Columbia, Canada.; 6 Vancouver Prostate Centre, Department of Urologic Sciences, University of British Columbia, Vancouver, British Columbia, Canada.; 7 Robert H. Lurie Comprehensive Cancer Center, Northwestern University Feinberg School of Medicine, Chicago, Illinois, USA.; 8 Department of Chemistry, Northwestern University, Evanston, Illinois, USA.; 9 Department of Medical Oncology, Dana Farber Cancer Institute, Boston, Massachusetts, USA.; 10 Department of Urology, University of Washington, Seattle, Washington, USA.; 11 Department of Human Genetics and; 12 Winship Cancer Institute, Emory University School of Medicine, Atlanta, Georgia, USA.; 13 Department of Biochemistry and Molecular Genetics, Northwestern University, Chicago, Illinois, USA.

**Keywords:** Oncology, Cancer, Cell cycle

## Abstract

CXCR7 is an atypical chemokine receptor that recruits β-arrestin (ARRB2) and internalizes into clathrin-coated intracellular vesicles where the complex acts as a scaffold for cytoplasmic kinase assembly and signal transduction. Here, we report that CXCR7 was elevated in the majority of prostate cancer (PCa) cases with neuroendocrine features (NEPC). CXCR7 markedly induced mitotic spindle and cell cycle gene expression. Mechanistically, we identified Aurora Kinase A (AURKA), a key regulator of mitosis, as a novel target that was bound and activated by the CXCR7-ARRB2 complex. CXCR7 interacted with proteins associated with microtubules and golgi, and, as such, the CXCR7-ARRB2-containing vesicles trafficked along the microtubules to the pericentrosomal golgi apparatus, where the complex interacted with AURKA. Accordingly, CXCR7 promoted PCa cell proliferation and tumor growth, which was mitigated by AURKA inhibition. In summary, our study reveals a critical role of CXCR7-ARRB2 in interacting and activating AURKA, which can be targeted by AURKA inhibitors to benefit a subset of patients with NEPC.

## Graphical abstract

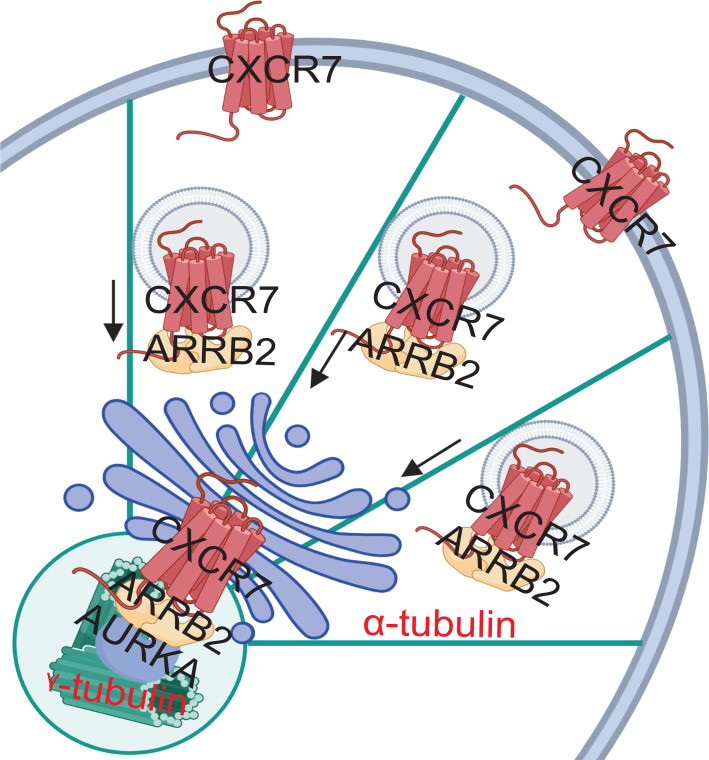

## Introduction

Prostate cancer (PCa) is the most commonly diagnosed cancer in the United States and the second leading cause of cancer-associated mortalities among males (1). Despite the overall decline in PCa mortalities that can be attributed to advancements in early diagnosis and targeted therapies, the incidence of metastatic PCa has been on the rise over the last decade (2, 3). Androgen receptor (AR) is a key driver of PCa development and progression, and androgen-deprivation therapy (ADT) is a mainstay of systemic treatments for nearly all patients with metastatic PCa (4). Unfortunately, the majority of patients relapse within 2–3 years of ADT with more aggressive, castration-resistant PCa (CRPC) (5). CRPC predominantly reactivates AR signaling through AR amplification, constitutive activation of AR or its splice variants, or adapting intratumoral de novo steroid biosynthesis (5–7). Thus, second-generation AR pathway inhibitors (ARPi), such as enzalutamide, have been shown to extend CRPC patient survival for several months (5). However, some CRPC tumors abandon AR signaling altogether, becoming AR^–^with neuroendocrine features (NEPC) (5, 6, 8). Therapy-induced NEPC is thought to be driven by linear plasticity, which causes loss of luminal and gain of neuroendocrine markers (9). Both de novo and therapy-induced NEPC are fast-proliferating tumors that quickly spread beyond the primary location (10). The rapid growth of NEPC can be attributed to the loss of oncosuppressors such as p53, PTEN, and RB1, with concomitant upregulation of cell cycle drivers including MYCN, PLK1, Cyclin D1, and AURKA (7, 11, 12). NEPC is a lethal disease whose treatment is currently limited to aggressive chemotherapy with docetaxel, etoposide, and platinum-based agents (10). Thus, there is an urgent need for effective targeted therapies.

CXCR7 is an atypical chemokine receptor that has been shown to drive cell proliferation in a number of aggressive tumors, such as bladder, breast carcinoma, and glioma (13–16). CXCR7 is a guanine nucleotide binding protein–coupled (G protein-coupled) receptor (GPCR) that recruits and interacts with cytoplasmic β-arrestin (ARRB2). The CXCR7-ARRB2 complexes internalize into clathrin-coated pits and endosomes, where they serve as an activating protein scaffold for cytoplasmic kinases, such as mitogen-activated protein kinase (MAPK) (17–19). We and others have previously shown that CXCR7 is a target of AR-mediated transcriptional repression, and, as such, CXCR7 is upregulated in PCa following treatment by ADT or ARPi, such as enzalutamide (20, 21). We illustrated that CXCR7 promotes CRPC resistance to enzalutamide by activating MAPK/ERK signaling (20). Further, emerging data suggest that CXCR7 signaling also regulates AKT, EGFR, and JAK2/STAT3 in prostate or breast cancers (16, 21–23). However, CXCR7 expression, function, and downstream pathways in NEPC have not been investigated.

Aurora Kinase A (AURKA) is a mitotic serine/threonine-protein kinase that plays an essential role in cell cycle regulation. During the G2 phase of mitosis, AURKA controls centrosome maturation as it phosphorylates TACC3 and targets pTACC3 to the mitotic centrosome (24). Phosphorylated TACC3 organizes microtubule nucleation and polymerization from the mitotic centrosome, a step critical for proper mitotic spindle assembly and cytokinesis (25). While the loss of RB1 and p53 is common in several treatment-resistant cancers, including NEPC (12), AURKA is frequently upregulated in NEPC tumors (9). Interestingly, AURKA inhibition is synthetically lethal with RB1 and p53 loss, suggesting that AURKA plays a critical role in pushing through the cell cycle in these tumors (26, 27). A Phase II clinical trial of the AURKA inhibitor alisertib for advanced PCa patients showed significant clinical benefit in a subset of 4 patients that expressed high levels of AURKA, although the study, overall, did not meet its primary endpoint due to drug toxicity and patient heterogeneity (28).

Here, we report a dominant role of CXCR7 in regulating downstream genes involved in the mitotic spindle and cell cycle progression. Combining genomic, proteomic, and biochemical techniques, we identified AURKA as a target kinase that was bound and activated by the CXCR7-ARRB2 complex. Interestingly, we found that the CXCR7-ARRB2 complex on the endosome membrane was transported along the microtubule to the pericentrosomal Golgi apparatus, where it interacted with and activated AURKA. Targeting of AURKA successfully abolished CXCR7-driven PCa cell proliferation in vitro and xenograft tumor growth in vivo. Taken together, our data suggest AURKA targeting as a promising therapeutic approach for advanced PCa with high CXCR7 expression.

## Results

### CXCR7 is upregulated in neuroendocrine PCa.

**Figure 1:**
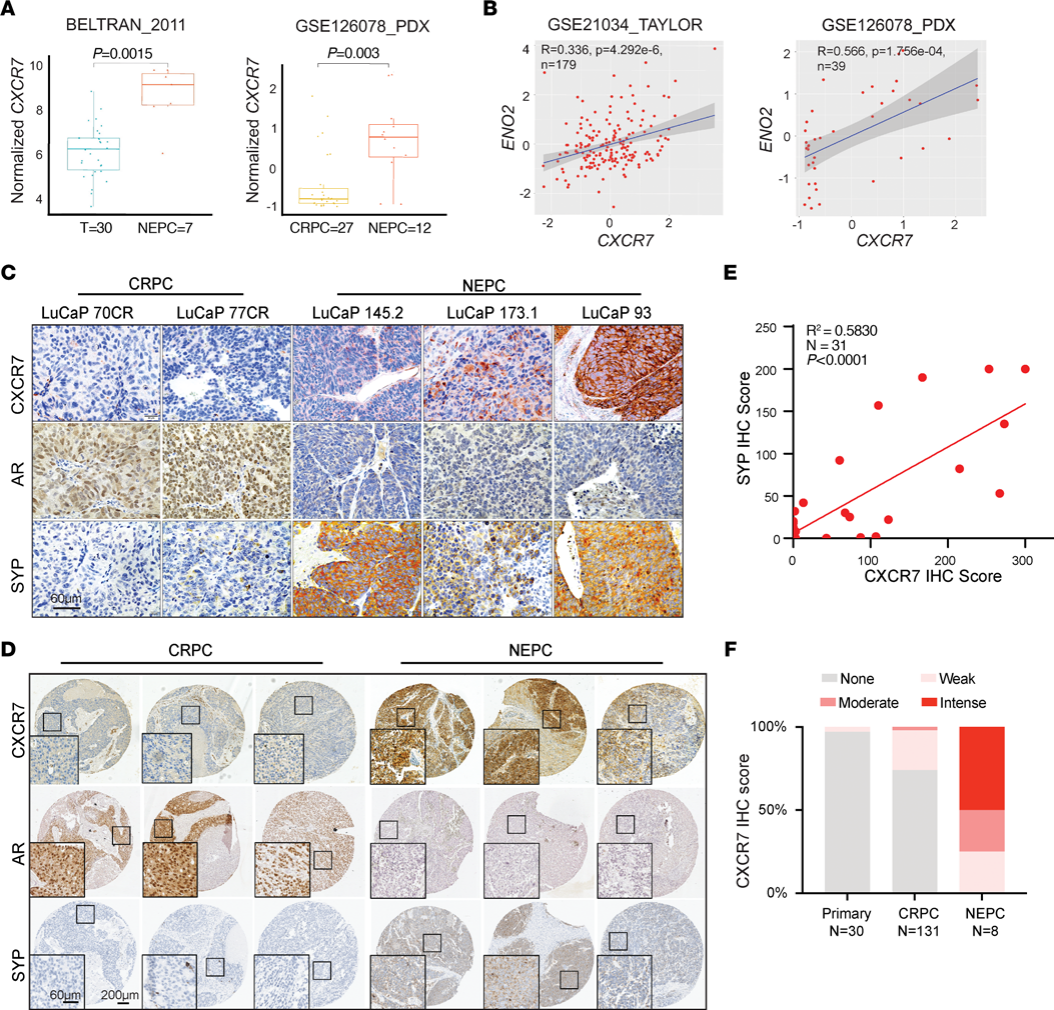
CXCR7 is upregulated in neuroendocrine PCa. (**A**) The box plots show that *CXCR7* is significantly upregulated in NEPC tumors. *CXCR7* (*ACKR3*) expression (mRNA) was queried from the data sets indicated. (**B**) Scatter plots show a significant correlation between *CXCR7* and *ENO2* in the PCa patient data sets. The dark grey area indicates the 95% CI, X- and Y-axes show normalized expression. Statistical analysis is based on linear regression. (**C**) Representative IHC staining of CXCR7, AR, and SYP in selected CRPC or NEPC LuCaP PDX tumors. Scale bar: 60 μm. (**D**) Tissue microarray constructed with clinical tumor samples was subjected to IHC staining with anti-CXCR7 (RnD, 11G8), anti-AR (AR-N Biogenex, MU256-UC), and anti-SYP (Santa Cruz, SC-17750) antibodies. Representative images of 3 independent CRPC or NEPC tumors are shown. Scale bars: 60 μm (inset); 200 μm (larger image). (**E**) Correlation between CXCR7 and SYP IHC staining scores in TMAs. Every dot represents the average intensity score of 3 cores for each tumor. A total of 31 tumors were analyzed. *P* < 0.001 by linear regression. (**F**) Quantification of CXCR7 IHC intensity scores in primary PCa, CRPC, and NEPC samples. The Y-axis shows the percentage of tumors with none (0; gray), weak (1; light pink), moderate (2; dark pink), and intense (3; red) IHC scores for each category.

We have previously reported that *CXCR7* is a direct target of AR-mediated transcriptional repression (20) and, as such, it is upregulated following enzalutamide treatment. To determine whether CXCR7 expression further increases as enzalutamide resistant PCa progresses to NEPC with a gain of neuroendocrine features and/or loss of AR expression (7), we examined several gene expression data sets of human PCa samples. Such data showed that *CXCR7* mRNA levels were significantly induced in NEPC compared with primary PCa or CRPC (Figure 1A and Supplemental Figure 1A; supplemental material available online with this article; https://doi.org/10.1172/JCI166248DS1). Further, *CXCR7* expression positively correlated with the expression of NEPC markers, such as *ENO2*, *CHGB*, and *SYP*, across PCa samples of multiple independent PCa patient cohorts (Figure 1B and Supplemental Figure 1, B and C). Moreover, we observed a highly significant and positive correlation between *CXCR7* expression and the proliferation marker *MKI67*, indicating its potential to regulate cell growth (Supplemental Figure 1D).

To confirm alterations in CXCR7 expression at the protein level, we performed an IHC analysis of several LuCaP models of patient-derived xenografts (PDX) (29). The data showed that CRPC tumors, which often express high levels of AR, had low CXCR7 expression. By contrast, NEPC LuCaP lines that are devoid of AR but stained strong for SYP showed a much higher protein level of CXCR7 (Figure 1C). Similarly, increased expression of CXCR7 was also observed in NEPC PDX tumors that were independently developed by the Living Tumor Laboratory (LTL) (30) (Supplemental Figure 1E).

To further validate our observation in clinical samples, we investigated a set of tissue microarrays containing clinical CRPC and NEPC tumor sections. We confirmed that CXCR7 IHC staining was substantially higher in NEPC tumors that stained negatively for AR but positively for SYP (Figure 1D). Further, analysis of IHC staining across all tumor sections showed a positive correlation between the intensity scores of CXCR7 and SYP, CXCR7, and CHGA (Figure 1E and Supplemental Figure 1F). To evaluate the percentage of tumors with high CXCR7 expression at varying stages of PCa progression, we analyzed CXCR7 IHC staining intensity in primary tumors (*n* = 30), CRPC (*n* = 131, using samples from Li et al. (20) and the current study), and NEPC (*n* = 8). We noticed that approximately 50% of NEPC tumors showed intense CXCR7 staining and another 25% of NEPC tumors expressed a moderate amount of CXCR7 (Figure 1F). By contrast, the vast majority of primary PCa were negative for CXCR7, and only a small portion (30%) of CRPC had moderate CXCR7 expression. Taken together, our results identify CXCR7 as a gene with an NEPC-specific expression profile.

### CXCR7 promotes mitotic spindle and cell cycle processes.

**Figure 2:**
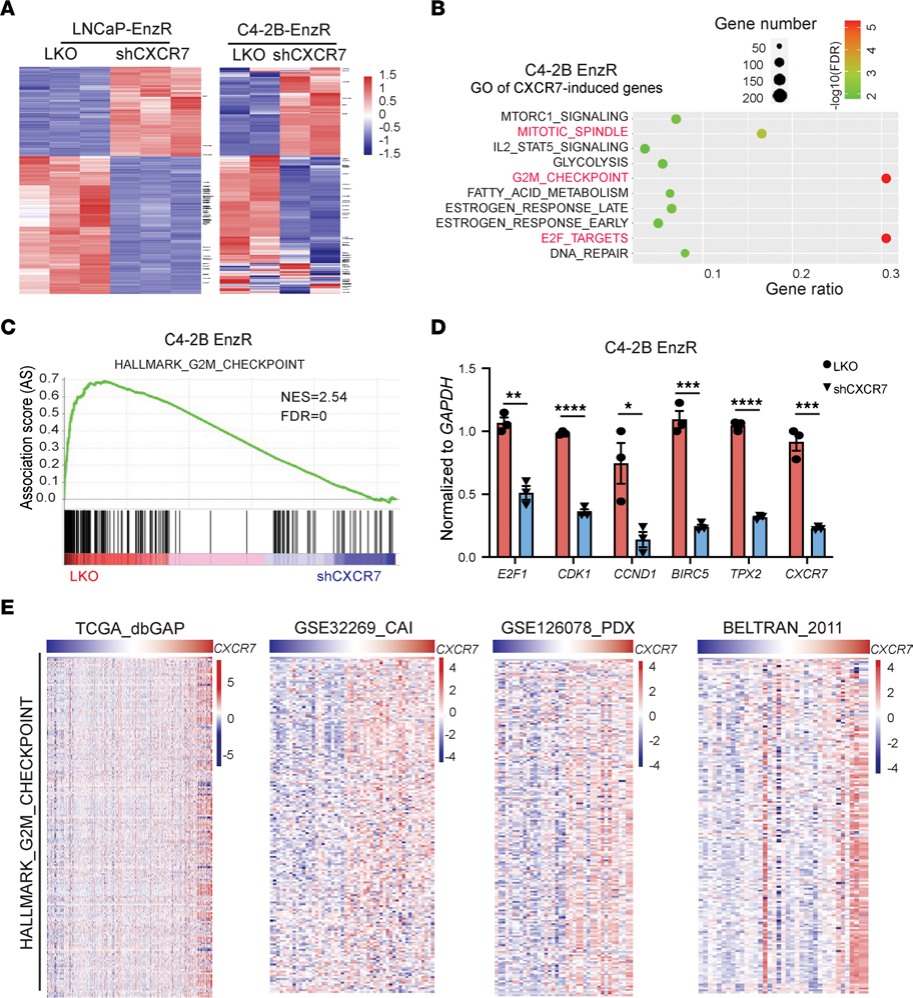
CXCR7 promotes mitotic spindle and cell cycle processes. (**A**) Heatmaps showing differentially expressed genes between control (LKO) and *CXCR7* KD (shCXCR7) LNCaP-EnzR cells by triplicate RNA-Seq analyses. Their expression in RNA-Seq data of duplicate C4-2B-EnzR cells is also shown. Cell cycle genes are listed on the right of the heatmaps. (**B**) GO analysis of *CXCR7*-induced genes in C4-2B-EnzR cells identified molecular concepts involved in the mitotic spindle and cell cycle. FDR, false discovery rate. (**C**) Gene Set Enrichment Analysis (GSEA) revealed that HALLMARK_G2M_CHECKPOINT molecular signature genes are enriched for down regulation upon *CXCR7* KD in C4-2B-EnzR cells. NES, normalized enrichment score. (**D**) qRT-PCR analyses of cell cycle genes in control and *CXCR7* KD C4-2B-EnzR cells. Data were normalized to *GAPDH* (mean ± SEM, *n* =3). *P <0.05, **P <0.01,***P <0.001, ****P <0.0001 between control versus KD cells, by 2-tailed unpaired Student’s *t* test. (**E**) Heatmap view of the HALLMARK_G2M_CHECKPOINT signature genes in the indicated PCa patient data sets with samples ordered by *CXCR7* level (top row).

To characterize the downstream molecular pathways of *CXCR7* in advanced PCa, we performed *CXCR7* knockdown (KD) in LNCaP-EnzR and C4-2B-EnzR cells, which are, respectively, LNCaP and C4-2B cells that have developed resistance to enzalutamide after several months of exposure. These stable cell lines showed increased expression levels of *CXCR7* as well as NE markers *ENO2* and *SYP*, mimicking clinical situations where patients with advanced PCa develop resistance to ARPi treatment and start to gain NE features (Supplemental Figure 2A). Triplicate RNA-Seq analyses of LNCaP-EnzR cells with control or *CXCR7* KD identified 335 and 516 genes that were respectively induced and repressed upon *CXCR7* depletion with at least 2-fold changes and an adjusted FDR of less than 0.001 (Figure 2A). This regulation was confirmed in duplicate RNA-Seq of control and *CXCR7* KD C4-2B-EnzR cells (GSE199274).

Gene Ontology (GO) analysis revealed that *CXCR7*-induced genes were strongly enriched in molecular pathways belonging to mitotic spindle assembly and G2/M checkpoint, both of which are critically regulated by AURKA and other centrosome proteins (31) (Figure 2B). By contrast, *CXCR7*-repressed genes were involved in myogenesis and epithelial-to-mesenchymal transition (Supplemental Figure 2B). Gene-Set Enrichment Analysis (GSEA) confirmed the upregulation of G2M checkpoint genes in control versus *CXCR7*-depleted C4-2B-EnzR and LNCaP-EnzR cells (Figure 2C and Supplemental Figure 2C). Further, quantitative real-time PCR (qRT-PCR) analysis confirmed decreased expression of a set of key cell cycle regulators, including *E2F1*, *CDK1*, and *CCND1*, following *CXCR7* KD (Figure 2D). To evaluate the relevance of *CXCR7* regulation of cell proliferation in clinical samples, we examined gene expression in previously published PCa patient data sets (11, 32, 33). We observed that the signature genes of G2/M checkpoint and mitotic spindle pathways were upregulated in PCa tumors with higher *CXCR7* expression compared with those with low *CXCR7* (Figure 2E and Supplemental Figure 2D). These results support that CXCR7 is critical in regulating mitotic spindle and cell cycle processes.

### CXCR7 regulates AURKA signaling.

**Figure 3:**
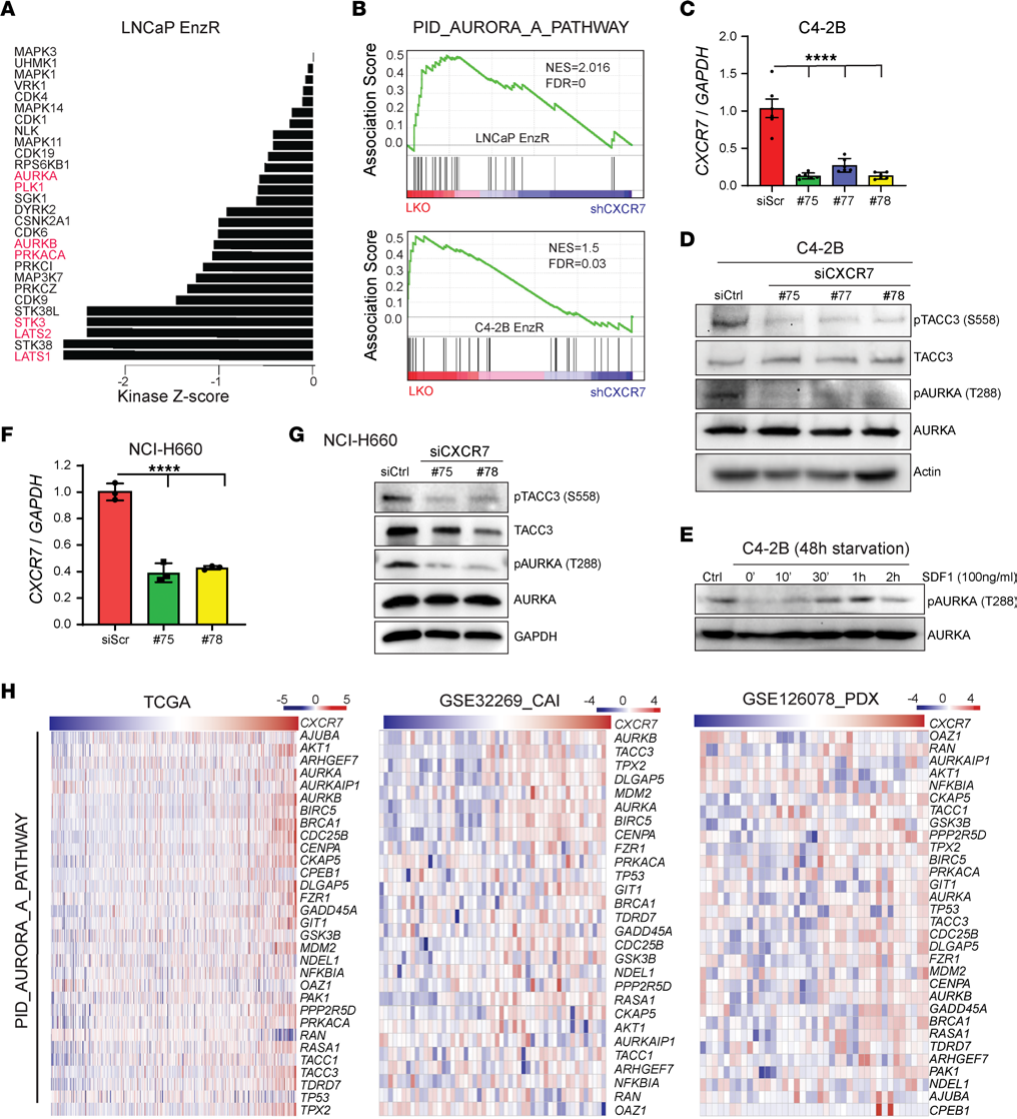
CXCR7 promotes AURKA signal transduction. (**A**) Control or CXCR7-KD LNCaP-EnzR cells were subjected to comprehensive phosphoproteome profiling. The array readings were analyzed with KSEA App, and a kinase score, representing changes in the kinase activity, was evaluated for each individual kinase. The plot shows kinases with a negative kinase score, indicating reduced activity upon *CXCR7* depletion. Red denotes kinases of the AURKA pathway. (**B**) GSEA showing that the PID_AURORA_A_PATHWAY molecular signature is enriched for down regulation upon *CXCR7* KD in LNCaP-EnzR (top) and C4-2B-EnzR (bottom) cells. NES, normalized enrichment score; FDR, false discovery rate. (**C** and **D**) C4-2B cells were treated with 3 independent *CXCR7*-targeting siRNAs (siCXCR7) or scrambled control (siCtrl) for 48 hours and then collected for qRT-PCR (**C**) and Western blot (**D**) analyses. qRT-PCR data were normalized to *GAPDH* and the control condition (mean ± SEM, *n* = 5). The statistical test is based on 1-way ANOVA paired with Dunnett’s multiple comparison test, ****P < 0.0001. (**E**) C4-2B cells were starved for 24 hours in serum-free media and stimulated with 100 ng/mL of recombinant SDF1 over a time course and resolved for protein analysis. Total AURKA was used as a loading control. (**F** and **G**) NCI-H660 cells were subjected to repeated siCXCR7 for 48 hours each and collected for analyses by qRT-PCR (**F**) and Western blot (**G**). qRT-PCR data were normalized to *GAPDH* and then the control condition (mean ± SEM, *n* = 3). The statistical test is based on 1-way ANOVA paired with Dunnett’s multiple comparison test, ****P < 0.0001. (**H**) Heatmap view of the PID_AURORA_A_PATHWAY molecular signature genes in the indicated PCa patient data sets. The samples were sorted by *CXCR7* expression from left to right.

CXCR7 is an atypical chemokine receptor that recruits ARRB2 to form a CXCR7-ARRB2 complex that internalizes into clathrin-coated vesicles and functions as a scaffold for the assembly and activation of cytoplasmic kinases (20). To identify the kinases whose activity is regulated by CXCR7-ARRB2, we analyzed CXCR7-KD LNCaP-EnzR cells using comprehensive phosphoproteomics, measuring changes in the entire kinome through relative quantification of phosphopeptides (34). The results showed that *CXCR7* KD decreased the phosphorylation of 661 unique peptides by at least 2-fold while increasing the phosphorylation of 524 unique peptides (Supplemental Table 2). Notably, data confirmed that *CXCR7* KD inhibited MAPKs, as we reported previously (20). To identify top inhibited kinases upon *CXCR7* depletion, we used the Kinase-Substrate Enrichment Analysis (KSEA), which evaluates the kinases’ activity based on the changes in the phosphorylation status of their identified substrates. Importantly, KSEA revealed that *CXCR7* depletion reduced a large number of kinases responsible for cell cycle progressions, such as cyclin-dependent kinases (CDKs), AURKA, and many AURKA-downstream kinases, including PLK1, AURKB, PRKACA, STK3, LATS1, and LATS2 (35–37) (Figure 3A).

To examine whether *CXCR7* KD indeed alters the AURKA signaling pathway, we performed GSEA analysis of the AURKA gene signature (PID_AURORA_A_PATHWAY) (38) and found that they were downregulated upon *CXCR7* depletion in LNCaP-EnzR as well as in C4-2B-EnzR cells (Figure 3B). To determine whether CXCR7 regulated AURKA activation, we used 3 independent siRNA to deplete *CXCR7* in C4-2B cells (Figure 3C). We observed a consistent decrease of AURKA self-phosphorylation at the T288 residue as well as the phosphorylation of its specific substrate, TACC3, at the S558 residue upon *CXCR7* KD (Figure 3D and Supplemental Figure 3A). Stromal cell-derived factor 1 (SDF1), also called CXCL12, present in the cell culture medium, is a cognate CXCR7 ligand that could enhance CXCR7 signaling in addition to its constitutive function (20). We found that serum starvation reduced AURKA phosphorylation, which was reactivated by adding back 100 ng/mL recombinant SDF1 (Figure 3E). Next, we sought to evaluate whether CXCR7 regulated AURKA signaling in NEPC cells. Importantly, we observed a drastic decrease in AURKA T288 and TACC3 S558 phosphorylation upon *CXCR7* KD in NCI-H660, in agreement with the C4-2B data, suggesting this as a general pathway in PCa (Figure 3, F and G). Finally, analyses of clinical PCa gene expression data sets show that AURKA-downstream signature genes were substantially upregulated in *CXCR7*-high tumors compared to *CXCR7*-low tumors (Figure 3H and Supplemental Figure 3, B and C). Collectively, these results indicate that CXCR7 induces AURKA phosphorylation and activates AURKA signal transduction.

### CXCR7-ARRB2 complex interacts with AURKA.

**Figure 4:**
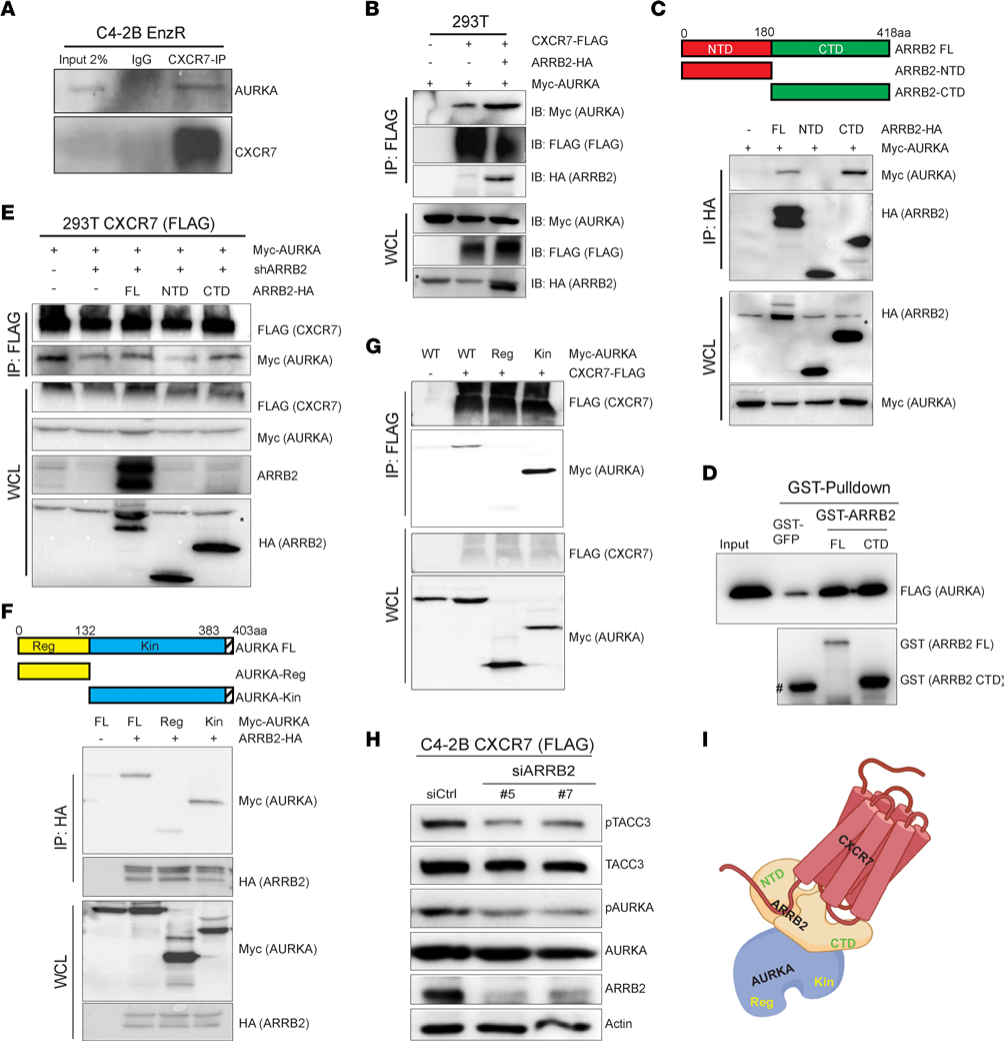
The CXCR7-ARRB2 protein complex interacts with AURKA. (**A**) The whole lysate of C4-2B-EnzR cells was subjected to anti-CXCR7 and IgG co-IP, followed by Western blot analyses. (**B**) ARRB2 increases CXCR7 and AURKA interaction. The 293T cells overexpressing Myc-AURKA, CXCR7-FLAG, and/or ARRB2-HA were subjected to co-IP with anti-FLAG antibodies, followed by Western blot. WCL, whole-cell lysate. (**C**) AURKA binds to the C-terminal domain (CTD) of ARRB2. 293T cells were cotransfected with Myc-AURKA and an empty vector, full-length (FL), N-terminal domain (NTD), or CTD of ARRB2. Co-IP was performed with an anti-HA antibody, followed by Western blot. (**D**) GST pull-down assay shows direct interaction between ARRB2 and AURKA. GST-ARRB2 FL, GST-ARRB2 CTD, or GST-GFP control proteins were incubated with FLAG-AURKA protein for 2 hours, separated by GSH-Sepharose, and resolved for Western blot. #, GST-GFP control band. (**E**) The CTD of ARRB2 restores CXCR7-AURKA interaction. The 293T-CXCR7 cells were transfected with the indicated plasmids and then subjected to co-IP using an anti-FLAG antibody, followed by Western blot analysis. (**F**) The kinase domain of AURKA binds to ARRB2. The 293T cells were cotransfected with ARRB2-HA and Myc-AURKA, FL, its regulatory (Reg), or kinase domain (Kin). Co-IP was performed with an anti-HA antibody, followed by WB analysis. (**G**) The kinase domain of AURKA forms a complex with CXCR7. CXCR7-FLAG construct was cotransfected in 293T cells with Myc-AURKA FL, regulatory domain (Reg), or kinase domain (Kin). Co-IP was performed with an anti-FLAG antibody, followed by Western blot. (**H**) ARRB2 KD decreases AURKA activation. C4-2B-CXCR7 cells were transfected with 2 independent siARRB2 for 48 hours and then analyzed by Western blot. Actin was used as a loading control. (**I**) A model depicting the interaction among the CXCR7-ARRB2-AURKA protein complex. Both the CTD and NTD of ARRB2 can interact with CXCR7, while only the CTD of ARRB2 binds to the kinase (Kin) domain of AURKA. The image was generated in BioRender. *, non-specific bands.

Since CXCR7 can activate the AURKA function, we hypothesized that CXCR7 interacts with the AURKA protein. To this end, we performed CXCR7 coimmunoprecipitation (co-IP) of C4-2B-EnzR cell lysates, which, indeed, showed coenrichment for endogenous AURKA (Figure 4A). We also tested the interaction between exogenous CXCR7 and AURKA by cotransfecting 293T cells with AURKA tagged with Myc at its N-terminus and CXCR7 tagged with FLAG at its C-terminus (Supplemental Figure 4A). Co-IP using anti-Flag (CXCR7) showed coenrichment of AURKA protein in the CXCR7-FLAG fraction of cell lysates, supporting the hypothesis that ectopic CXCR7 protein interacted with AURKA. To determine whether CXCR7-AURKA interaction involves ARRB2, which is known to complex with CXCR7 to form a scaffold for kinase activation (20), we sequentially cotransfected Myc-AURKA, CXCR7-FLAG, and ARRB2-HA into 293T cells. Co-IP using anti-FLAG (CXCR7) revealed that concomitant expression of exogenous ARRB2 greatly increased Myc-AURKA coenrichment with CXCR7 (Figure 4B), indicating that ARRB2 mediated AURKA-CXCR7 interaction. To map the ARRB2 domains that mediate its interaction with AURKA, we cloned N- and C-terminal domains (NTD and CTD) of ARRB2 (39) and cotransfected them with Myc-AURKA into 293T cells. Anti-HA (ARRB2) co-IP revealed that AURKA bound to the CTD of ARRB2 (Figure 4C). Moreover, we isolated GST-tagged ARRB2 full-length or CTD and performed a GST pull-down assay with recombinant AURKA. We observed that ARRB2 CTD was sufficient to bind AURKA directly, similar to the full-length ARRB2 (Figure 4D).

To further validate that ARRB2 mediates CXCR7 interaction with AURKA, we depleted endogenous ARRB2 in 293 cells with the expression of exogenous CXCR7-FLAG and Myc-AURKA. We found that ARRB2 depletion abolished the coenrichment of Myc-AURKA by CXCR7 co-IP. Critically, overexpression of full-length ARRB2, as well as its CTD, restored AURKA pull down by CXCR7 co-IP, while ARRB2-NTD cannot mediate CXCR7-AURKA interaction (Figure 4E). By contrast, it has been previously reported that both CTD and NTD of ARRB2 can bind to 7 transmembrane domain receptors (7TMR), such as CXCR7 (40). These results indicated that ARRB2-CTD was required to bridge the interaction between CXCR7 and AURKA proteins.

To determine which domain of AURKA interacts with ARRB2, we likewise cloned full-length AURKA and its N-terminal regulatory domain and C-terminal kinase domain constructs (41) (Figure 4E). We cotransfected full-length Myc-AURKA or its domain constructs together with ARRB2-HA into 293T cells. Co-IP with anti-HA (ARRB2) antibodies revealed that AURKA bound to ARRB2 primarily through its kinase domain (Figure 4F). Likewise, co-IP of CXCR7 showed coenrichment of full-length AURKA and the C-terminal kinase domain that were able to bind ARRB2, but not the ARRB2-disabled N-terminal regulatory domain of AURKA (Figure 4G). Further, co-IP with anti-HA antibodies in C4-2B cells with coexpression of ARRB2-HA and Myc-AURKA confirmed their interaction in PCa cells (Supplemental Figure 4B). We have now established that ARRB2 mediated CXCR7-AURKA protein interaction, so we asked if ARRB2 depletion abolished AURKA activation. Indeed, *ARRB2* KD using 2 independent siRNAs reduced the phosphorylation of AURKA (T288) and TACC3 (S558) in C4-2B cells (Figure 4H). Collectively, our data reveal a novel protein-protein complex wherein ARRB2-CTD binds to the kinase domain of AURKA to mediate its interaction with CXCR7, which binds to ARRB2 through both its CTD and NTD (Figure 4I).

### CXCR7, ARRB2, and AURKA colocalize at the pericentrosomal region.

**Figure 5:**
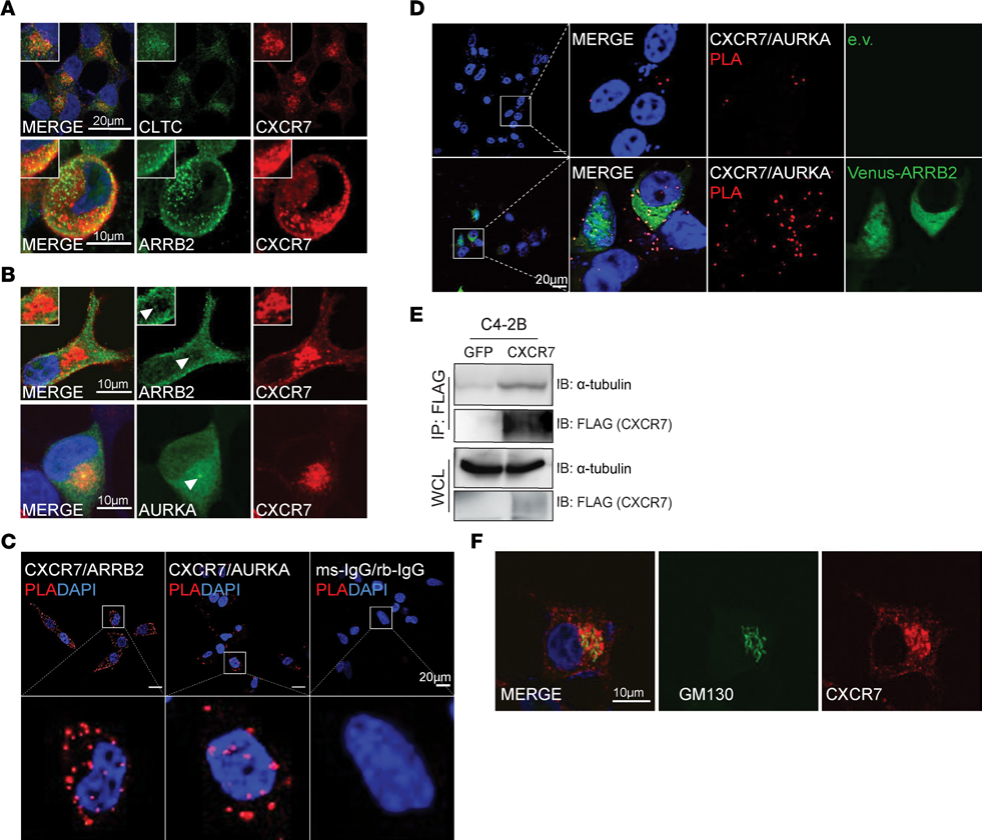
CXCR7, ARRB2, and AURKA colocalize at the pericentrosomal region. (**A**) C4-2B cells with stable CXCR7 overexpression were subjected to confocal imaging showing cytoplasmic colocalization of CXCR7 and clathrin heavy chain (CLTC, top) or ARRB2 (bottom). Scale bars: 20 μm (top); 10 μm (bottom).(**B**) Confocal IF images of C4-2B-CXCR7 cells show pericentrosomal localization of CXCR7 surrounding centrosomal ARRB2 (top) and AURKA (bottom). White arrowheads point to the centrosomes. (**C**) PLA showing molecular interactions between CXCR7 and ARRB2 (left) and between CXCR7 and AURKA (center) in C4-2B cells. PLA with control IgG antibodies was performed as a negative control (right). Scale bar: 20 μm. (**D**) C4-2B cells were transfected with an empty vector (e.v.; top row) or Venus-ARRB2 (bottom row). The cells were then subjected to PLA (3rd column), shown in the context of DAPI (1st–2nd column) and ARRB2 (4th column) signals. Scale bar: 20 μm. (**E**) CXCR7 interacts with α-tubulin. Control (GFP) or CXCR7-FLAG expressing C4-2B cells were subjected to co-IP by an anti-FLAG antibody, followed by Western blot. (**F**) Confocal IF imaging shows the accumulation of CXCR7 at the Golgi complex. C4-2B cells with stable CXCR7 overexpression were subjected to IF costaining for CXCR7 and GM130, a Golgi marker. Scale bar: 10 μm.

As a GPCR, membrane-bound CXCR7 interacts with cytosolic ARRB2, followed by endocytosis and intracellular internalization of the CXCR7-ARRB2 protein complex, which acts as a scaffold for cytoplasmic protein kinase assembly and substrate activation (19, 39, 42). AURKA regulates mitotic spindle assembly and concentrates on the centrosome, although nuclear and cytoplasmic AURKA has also been reported (43). Therefore, we asked in what cellular compartment the CXCR7-ARRB2 complex encounters with the AURKA protein. Toward this end, we first examined whether CXCR7 remained in the clathrin-coated endosomes and colocalized with ARRB2 in PCa cells. Immunofluorescence (IF) staining, followed by confocal microscopy, showed that CXCR7 was primarily internalized to the intracellular compartment (Figure 5A). IF costaining further confirmed that CXCR7 colocalized with clathrin heavy chain (CLTC) and ARRB2. Interestingly, we observed in a subset of cells that ARRB2 accumulated at centrosomes, where AURKA localizes, and was surrounded by a high density of CXCR7 at the pericentrosomal area (Figure 5B). To further confirm this, we performed IF colocalization analyses exploiting the 293T cells expressing Venus-tagged ARRB2. We observed a strong Venus fluorescent signal indicating high-density ARRB2 protein at the centrosomes marked by punctate AURKA staining, which is consistent with previous reports of ARRB2 targeting to the centrosomes in cycling cells (44) (Supplemental Figure 5A). Overall, these data suggest that AURKA interacts with CXCR7-ARRB2 at the centrosomal regions.

To further validate the interaction between endogenous CXCR7, ARRB2, and AURKA proteins in situ, we adapted the proximity ligation assays (PLA). First, we performed a PLA assay on C4-2B cells using CXCR7- and ARRB2-specific antibodies and detected a strong PLA signal confirming that CXCR and ARRB2 proteins were in close proximity, as expected (20). Critically, a PLA assay using anti-CXCR7 and anti-AURKA antibodies detected a similarly strong PLA signal indicating CXCR7 interaction with AURKA, while no PLA signal was detected in cells treated with isotype control antibodies (Figure 5C). Further, we attempted to use the PLA assay to investigate if ARRB2 could increase CXCR7 and AURKA colocalization. To this end, we overexpressed Venus-tagged ARRB2 in C4-2B cells and performed PLA. We, indeed, observed a significant increase in PLA speckles in cells with ARRB2 overexpression, indicating increased CXCR7 and AURKA protein interaction and, thus, colocalization (Figure 5D and Supplemental Figure 5B).

To understand the potential mechanisms for CXCR7 and AURKA protein colocalization and interaction, we next sought to perform unbiased profiling of CXCR7-interacting proteins in PCa cells. Mass spectrometry analysis of LNCaP-CXCR7 cells showed that CXCR7 interacted with a large number of microtubule-associated proteins such as TUBB4B and TUBA1C, vesicle-associated proteins such as CLTC and SEC22B, as well as many Golgi-associated proteins like GBF1, GPR89B, and GOLPH3 (Table 1 and Supplemental Table 3). Co-IP confirmed that CXCR7 bound to α-tubulin, a microtubule-building unit (Figure 5E), whereas IF costaining showed that CXCR7 colocalized with GM130, a marker of the Golgi apparatus (Figure 5F), which is spatially and functionally associated with the centrosomes (45). Taken together, these data support that CXCR7 interacts with microtubule- and Golgi-associated proteins and colocalizes with AURKA at the perinuclear area surrounding centrosomes, including the Golgi apparatus.

### CXCR7 is transported along the microtubules to the pericentrosomal Golgi apparatus.

**Figure 6:**
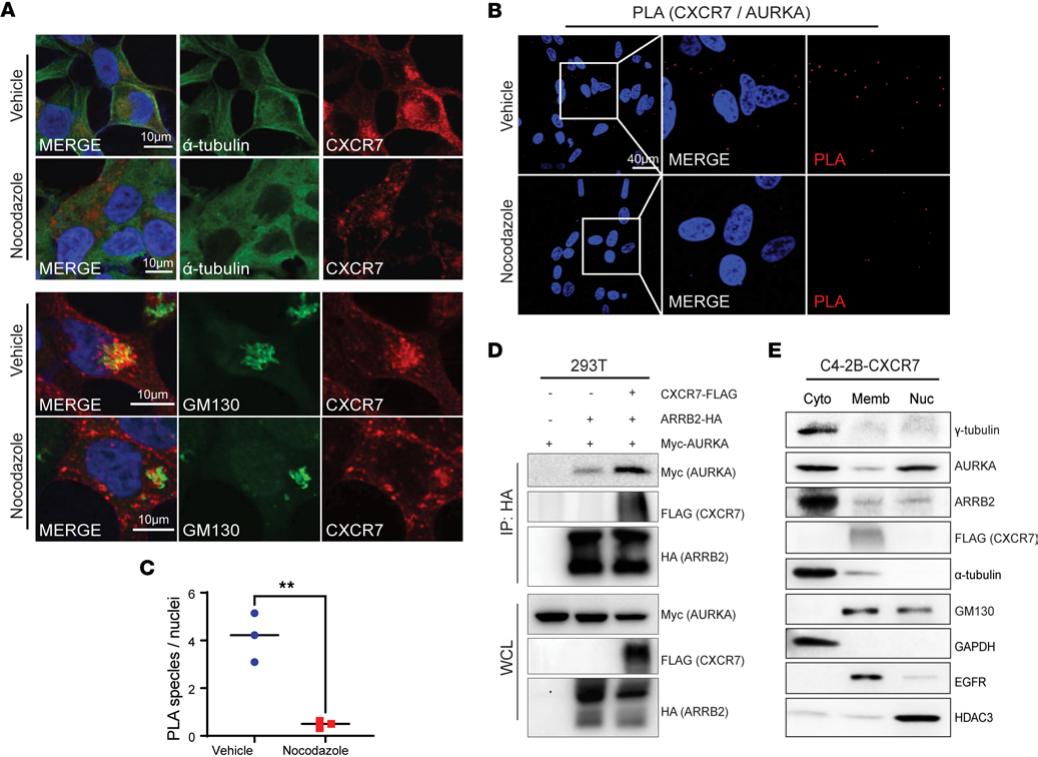
CXCR7 is transported along the microtubules to the pericentrosomal Golgi apparatus. (**A**) Microtubule destabilization impairs CXCR7 colocalization with α-tubulin and accumulation at the Golgi apparatus. C4-2B cells with stable CXCR7 overexpression were treated with either vehicle control or nocodazole, a microtubule polymerization inhibitor, at 10μg/mL for 1 hour and then subjected to IF costaining of CXCR7 and α-tubulin (top 2 rows) or GM130 (bottom 2 rows). Scale bar: 10 μm(**B** and **C**) In situ PLA of C4-2B cells that were pretreated with DMSO or 10 μg/mL of nocodazole for 1 hour shows decreased interaction between CXCR7 and AURKA in drug-treated cells, as shown by the red speckles (**B**) that were quantified in **C**. Scale bar: 40 μm The number of speckles was counted and normalized to the number of nuclei from 3 individual imaged field views (n=3, 2-tailed unpaired Student’s *t* test, **P <0.01). (**D**) CXCR7 enhances ARRB2-AURKA interaction. 293T cells were transiently transfected with a combination of indicated plasmids for 48 hours and then subjected to co-IP with anti-HA antibodies, followed by Western blot. (**E**) CXCR7 cofractionates with membrane, ARRB2, AURKA, and α-tubulin**.** C4-2B cells with stable CXCR7 overexpression were subjected to subcellular fractionation for cytoplasmic (Cyto), membrane (Memb), and nuclear (Nuc) fractions. The purity of the fractionation was validated by GAPDH (Cyto), EGFR (Memb), and HDAC3 (Nuc) markers.

As membrane CXCR7-ARRB2 is known to get internalized to intracellular vesicles, we attempted to understand how these vesicles move to the centrosomes. We hypothesized that CXCR7 moves to the Golgi complex through intracellular trafficking along the microtubules. Indeed, in vitro tubulin binding assay showed tubulin-dependent accumulation of CXCR7-FLAG in microtubule pellets, supporting that CXCR7 bound to microtubules (Supplemental Figure 6A). Further, we acutely treated PCa cells with nocodazole, an α-tubulin polymerization inhibitor. Significantly, confocal imaging demonstrated that nocodazole treatment depolymerized microtubules and decreased CXCR7 accumulation at the Golgi apparatus (Figure 6A). Similarly, acute nocodazole treatment reduced CXCR7 interaction with AURKA, evident through a significant decrease in PLA signal (Figure 6, B and C).

Since membrane CXCR7 interacts with ARRB2 to activate endocytosis and the internalization of the CXCR7-ARRB2 complex, we hypothesized that CXCR7 could increase ARRB2 interaction with AURKA. Indeed, co-IP showed that CXCR7 overexpression in 293T cells increased ARRB2-AURKA interaction (Figure 6D). To further validate their colocalization in the intracellular compartments, we isolated cytoplasmic, membranous, and nuclear fractions from C4-2B cells with stable CXCR7 overexpression. As expected, AURKA was found in the cytoplasm (including centrosome) as well as in the nuclei, as recently reported (46) (Figure 6E). Interestingly, most of ARRB2 was detected in the cytoplasmic fraction, similar to a centrosome marker γ-tubulin. By contrast, CXCR7 was localized exclusively to the membrane fraction, which includes the plasma membrane, intracellular vesicles, and membrane organelles, where it cofractionated with a portion of ARRB2, AURKA, α-tubulin, and Golgi marker GM130 (Figure 6E). Our data, therefore, suggested a model wherein the membrane-bound CXCR7 in intracellular vesicles interacted with ARRB2 and microtubule proteins such as α-tubulin and trafficked to the Golgi apparatus located at the pericentrosomal zone. Pericentrosomal CXCR7-ARRB2 surrounded and interacted with AURKA in the centrosome to facilitate protein complex integrity, likely to provide a scaffold for AURKA activation and, thus, cell cycle progression.

### CXCR7 increases PCa growth, which is abolished by AURKA inhibition.

**Figure 7:**
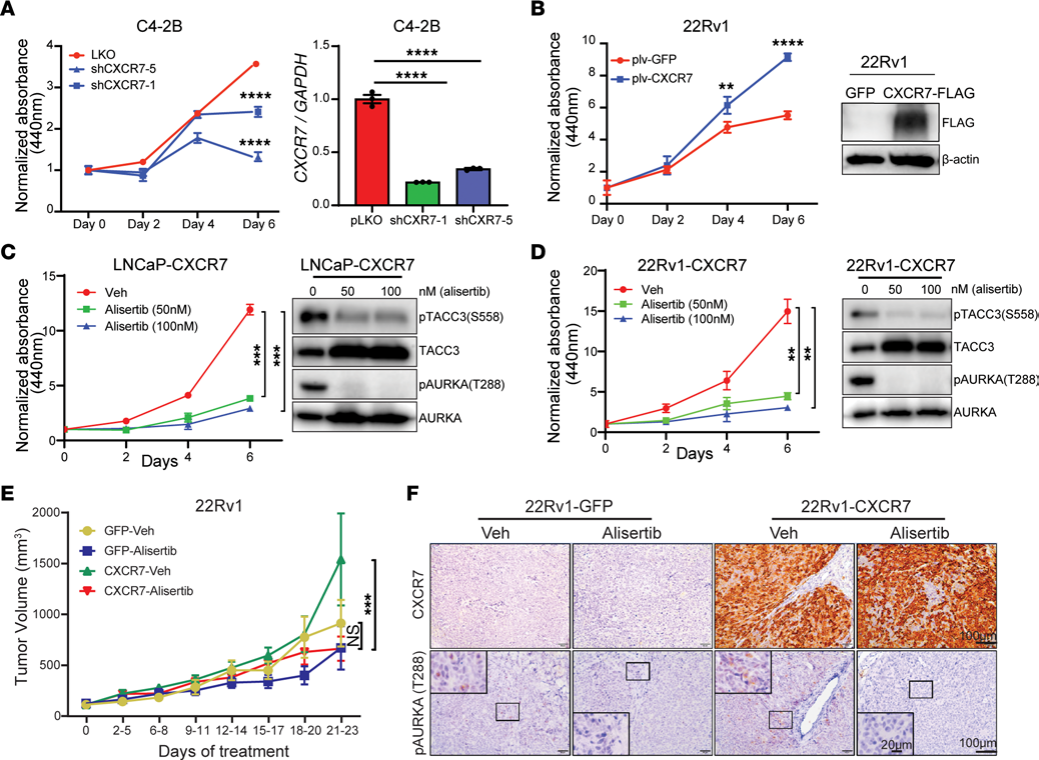
CXCR7 increases PCa growth, which is abolished by AURKA inhibition. (**A**) CXCR7 KD reduces C4-2B cell proliferation. WST1 assay was performed to measure cell proliferation. *CXCR7* KD was confirmed by qRT-PCR. Representative proliferation data from 3 repeated experiments are shown. The data were analyzed by 2-way ANOVA followed by Tukey-corrected multiple-comparison test (mean ± SD, *n* =3, ****P <0.0001). qRT-PCR data were normalized to *GAPDH* and then the control condition (mean ± SEM, *n* =3). Statistical test is based on 1-way ANOVA paired with Dunnett’s multiple comparison test,****P <0.0001. (**B**) CXCR7 overexpression, confirmed by Western blot, increases 22Rv1 cell proliferation measured by WST-1 assay. Representative proliferation data from 3 repeated experiments are shown here. The data were analyzed by 2-way ANOVA followed by Bonferroni-corrected multiple-comparison test (mean ± SD, *n* =3, **P <0.01, ****P <0.0001). (**C** and **D**) AURKA inhibitor decreases CXCR7-driven cell proliferation in LNCaP (**C**) and 22Rv1 (**D**) measured by WST-1 assay. Representative proliferation data from 3 repeated experiments were analyzed by 2-way ANOVA combined with Tukey-corrected multiple-comparison test (mean ± SD, n=3, **P<0.01 ***P<0.001). Western blot confirms a reduction of AURKA and TACC3 phosphorylation under alisertib treatment. (**E**) AURKA-targeting delays CXCR7-driven tumor growth in vivo. NSG mice were injected s.c. with 1 × 10^6^ of 22Rv1-GFP control or 22Rv1-CXCR7 cells. Tumor size (mm^3^) was monitored by caliper measurements twice a week. Once tumors reached 100 mm^3^, mice were randomized to receive either vehicle or alisertib (30 mg/kg) once a day for 21 days for a total of 4 treatment groups, *n* =4 (GFP-veh), *n* =5 (GFP-alisertib), *n* =5 (CXCR7-veh), *n* =5 (CXCR7-alisertib). Tumor growth data are shown as mean ± SEM. The statistical test is based on 2-way ANOVA combined with a Bonferroni’s-corrected multiple-comparison test (***P <0.001). (**F**) IHC staining of CXCR7 and pAURKA (T288) of representative tumor samples collected at the endpoint. Scale bars: 20 μm (inset); 100 μm (larger image).

Since CXCR7 promoted AURKA signaling and cell cycle gene expression, we attempted to determine whether CXCR7 induced PCa cell proliferation. We first evaluated the levels of endogenous CXCR7 using flow cytometry and observed that CXCR7 expression is the highest in C4-2B, medium in LNCaP, and the lowest in 22Rv1 cells (Supplemental Figure 7A). We then depleted *CXCR7* in C4-2B cells with 2 independent shRNAs and observed that *CXCR7* KD significantly decreased C4-2B cell proliferation (Figure 7A). A similar growth-inhibitory effect was observed in LNCaP cells with *CXCR7* KD (Supplemental Figure 7B). On the other hand, overexpression of ectopic CXCR7 significantly increased cell proliferation in 22Rv1 (Figure 7B) and LNCaP cells (Supplemental Figure 7C). These data supported CXCR7 as a promising therapeutic target in advanced PCa. However, there are currently no CXCR7-specific inhibitors that are clinically available (47). To test the efficacy of CXCR7 inhibition using pharmacological inhibitors, we exploited alisertib, an AURKA inhibitor that has shown significant clinical benefit in a subset of patients with advanced PCa suggestive of AURKA and N-Myc overactivity in a recent clinical trial (28). To test this, we performed WST cell proliferation assays of LNCaP and 22Rv1 cells and observed that alisertib abolished CXCR7-induced cell growth with concomitant suppression of AURKA activities (Figure 7, C and D). The effect of alisertib was much smaller in control LNCaP-GFP and 22Rv1-GFP cells that have lower CXCR7 expression (Supplemental Figure 7, D and E).

Next, we sought to investigate the efficacy of alisertib in targeting CXCR7-driven tumor growth in vivo. To this end, we inoculated 22Rv1 cells subcutaneously into the dorsal flank of the NSG mice. Once tumors reached 100 mm^3^, mice were randomized to receive either vehicle or 30 mg/kg of alisertib treatment once daily for 21 days. We observed that 22Rv1-CXCR7 cells developed tumors much faster compared with 22Rv1-GFP control cells, as expected. Importantly, treatment with alisertib significantly inhibited 22Rv1-CXCR7 tumor growth, and, to a much lesser extent, the 22Rv1-GFP tumors (Figure 7E). At the endpoint, we subjected study tumors for IHC analysis, which confirmed CXCR7 overexpression in the 22Rv1-CXCR7 tumors compared with the 22Rv1-GFP tumors. Accordingly, the AURKA phosphorylation level was elevated in CXCR7-overexpressing cells, and it was abolished following alisertib treatment (Figure 7F). Overall, these data suggest that CXCR7 upregulation in late-stage PCa provides an important mechanism for AURKA overactivity, which may be targeted using pharmacological inhibitors of AURKA.

## Discussion

CXCR7 is a scavenger chemokine receptor of CXCL12 that has been found to be upregulated in PCa (20, 22, 48–50). Over the years, several mechanisms for CXCR7 upregulation in advanced PCa have been identified. For example, CXCR7 is transcriptionally repressed by HIC1 (hypermethylated in cancer 1) or AR (20, 51), and, as such, loss of HIC1 or anti-AR therapy increases CXCR7 expression levels (20, 51, 52). Further, CXCR7 is reportedly induced by deletion of PTEN — through the upregulation of the transcription factor RUNX2 (53) — or upregulation of the precancerous cytokine IL-8 (22). We have previously observed CXCR7 upregulation in PCa following anti-AR treatment with enzalutamide (20). Here, we show that CXCR7 expression is further increased in the later-stage CRPC subtype, NEPC, which often harbors PTEN deletion, AR loss, and IL-8 upregulation (9, 54). Functionally, CXCR7 has been associated with increased adhesion and invasion, as well as cell cycle progression and proliferation (22). In the present study, we performed genome-wide expression profiling of PCa cells with *CXCR7* KD and reported that the primary role of CXCR7 was to regulate downstream genes involved in cell proliferation and cell cycle progression. We further identified AURKA, which is also highly expressed in NEPC, as a critical mediator of the CXCR7 function. We demonstrated that CXCR7 regulated AURKA phosphorylation, similar to previously reported EGFR, ERK, and AKT phosphorylation by CXCR7 (20–22). Overall, we think that CXCR7 promotes the cancer cell cycle and proliferation, likely through the regulation of a cohort of cytoplasmic kinases.

CXCR7 belongs to a large family of GPCRs, which feature an extracellular ligand-binding region, 7 transmembrane domains, and an intracellular region that interacts with cytoplasmic proteins. CXCR7 is an atypical GPCR in that it does not interact with G proteins (19, 55, 56). Instead, CXCR7 binds to ARRB2, which triggers CXCR7 internalization by engaging with clathrin-coated membrane pits (57). The internalized CXCR7 can be either recycled to the plasma membrane or retained in the cytoplasm to transduce ARRB2-dependent downstream signaling (58). Studies have shown that certain GPCR-containing vesicles accumulate at the perinuclear zone, interacting with the endoplasmic reticulum and trans-Golgi complex. Consistent with this notion, our mass spectrometry analyses revealed CXCR7 interaction with many proteins of the endosomes, endoplasmic reticulum, and Golgi, and IF costaining confirmed CXCR7 colocalization of the Golgi apparatus. In addition, CXCR7 showed strong interaction with α- and β-tubulin proteins. Our data suggested that CXCR7-ARRB2–containing vesicles trafficked along the microtubules to the pericentrosomal area, where they accumulated and interacted with centrosomal AURKA and enhanced AURKA phosphorylation and signaling. Accordingly, we found ARRB2 accumulation at the centrosome of PCa cells, consistent with earlier studies showing ARRB2 association with the centrosome to regulate its function (59). Of note, CXCR7 and AURUKA are also present in other areas of the cytoplasm, where they could also interact. Altogether, our data support the notion that the CXCR7-ARRB2 protein complex serves as a scaffold and orchestrates a cytoplasmic kinase/substrate–activating interaction and provides additional evidence for intracellular trafficking of the endosomal CXCR7-ARRB2**.**

Since CXCR7 has been recognized as a marker for aggressive cancer there have been numerous attempts to develop CXCR7 inhibitors that could block ARRB2 recruitment. The early effort produced a small molecule inhibitor, CCX771, which was thought to be a CXCR7 antagonist. It has been used in multiple studies as a therapeutic agent targeting CXCR7 and showed promise in combination with enzalutamide (49, 60–63). Surprisingly, CCX771 was later found to stimulate ARRB2 recruitment with even greater potency than many of the endogenous chemokine ligands, thus acting as a bona fide agonist (64). In fact, the nature of the CXCR7 protein structure renders it activation-prone by a number of endogenous ligands and exogenous agonists or antagonists (57, 65). Moreover, CXCR7 has ligand-independent activity evident through baseline ARRB2 recruitment and receptor internalization (20, 42). These characteristics pose a major challenge to the development of CXCR7 antagonists.

An alternative and practical approach to target the oncogene is to block its key downstream signaling. We have previously shown that MAPK inhibitors resensitize CXCR7^+^ CRPC to enzalutamide (20). Others have demonstrated that JAK2/STAT1 inhibition in combination with enzalutamide decreases the CXCR7-driven CRPC tumor growth (63). In the current work, we identify AURKA as a major mediator of CXCR7-driven PCa and show that AURKA inhibition reduces tumor growth. Although AURKA-targeting with alisertib in patients with NEPC showed that a handful of participating patients reached significant clinical benefit (28), the clinical trial failed to reach the primary endpoint due to toxicities associated with alisertib. New AURKA inhibitors with better biosafety profiles are needed and may be useful for the treatment of CXCR7-driven PCa. In addition, our mechanistic data showing CXCR7 trafficking along microtubules to interact with and activate AURKA suggest that tubulin-targeting drugs, such as docetaxel and cabazitaxel, might disrupt CXCR7-mediated AURKA activation and thus act in synergy with AURKA inhibitors. Indeed, several clinical trials have reported the promising efficacy of alisertib in combination with paclitaxel in advanced breast cancer and high-grade neuroendocrine tumors (66, 67). Similar clinical trials in advanced PCa and/or NEPC patients are warranted.

We acknowledge that the substantial body of mechanistic experiments in the present study was done in non-NEPC cell lines, such as C4-2B, LNCaP, and 22Rv1, which represent a limitation of the study. Although we only confirmed some key experiments in NCI-H660 NEPC cell line, we suggest that the described mechanism of CXCR7-ARRB2-directed regulation of AURKA activation is cell type-independent and could be prominent in NEPC, where there is a cooperative upregulation of both CXCR7 and AURKA expression.

## Methods

### Constructs, transfection, and lentiviral infection.

Plvx-CXCR7-FLAG and pDest-Myc-AURKA were cloned with Gateway LR Clonase II kit (Invitrogen). pCDN3.1-ARRB2-HA have been previously described (20). pCDN3.1-Venus ARRB2 was a gift from Vsevolod Gurevich (Vanderbilt University, Nashville, TN, USA). pCDN3.1-ARRB2-C-terminal domain-HA and pCDN3.1-ARRB2-N-terminal domain-HA constructs, pDest-Myc-AURKA-regulatory domain and pDest-Myc-AURKA-kinase domain, pGEX5-GST-ARRB2, and pGEX5-GST-ARRB2 C-terminal domain were cloned with In-Fusion HD cloning kit (Takara) and the cloning primers are listed in Supplemental Table 1.

To silence genes, we used siCXCR7 (SASI_Hs01_000628-75, -77, -78; Sigma-Aldrich) and siARRB2 (J-007292-05, -07; Dharmacon), transfected with Lipofectamine RNAiMAX (Invitrogen). To transiently overexpress genes, PCa cells were transfected with plasmid DNA mixed with X-tremeGENE HP DNA transfection reagent (Roche), and HEK293T cells were transfected with plasmid DNA mixed with polyethylenimine (Polyscience). For stable knock downs we used shCXCR7 (TRCN0000014509, TRCN0000378566; Sigma-Aldrich) or shARRB2 (TRCN0000159482; Sigma-Aldrich). Stable overexpression and KD were reached by lentiviral infection. Lentiviral particles were produced in HEK293T cells transfected with a mixture of plasmid DNA, psPAX2, a virus packaging plasmid, and pMD_2_G, an envelope plasmid, mixed with the PEI. The culture medium containing the lentiviruses was collected 48 hours after transfection and filtered through a 0.45 μm filter to remove cell debris. The filtered particles were added to the cell cultures in the presence of polybrene (8 μg/mL) and selected with puromycin (Gibco).

### RNA isolation, quantitative RT-PCR, and RNA sequencing.

Total RNA was isolated from cells with NucleoSpin RNA isolation kit (Takara). ReverTra Ace  qPCR RT Master Mix kit (Toyobo) was used for RNA reverse transcription. The qRT-PCR reaction was run with 2 × universal SYBR green fast qPCR mix (Abclonal) on StepOnePlus real-time PCR system (Applied Biosystems). Results were analyzed using StepOne Software v2.1 (Applied Biosystems), and relative expression of mRNA was determined using *GAPDH* as the loading control. qRT-PCR data were obtained in triplicate. PCR primers used in this study are listed in Supplemental Table 1. For RNA-Seq, total RNA was isolated as described above. RNA-Seq libraries were prepared from 0.5 μg high-quality DNA-free RNA using NEBNext ultra RNA library prep kit, according to the manufacturer’s instructions. The libraries that passed quality control, having equal size distribution between 250–400 bp, no adapter contamination peaks, and no degradation peaks, were quantified using the library quantification kit from Illumina (Kapa Biosystems). Libraries were pooled to a final concentration of 10 nM and sequenced single-end using the Illumina HiSeq 4000.

### Western blotting, coimmunoprecipitation, and protein fractionation.

Detailed procedure is described in the supplemental materials. Total protein lysate cells were washed once in PBS and lysed by 5 minutes boiling in 1 × SDS lysis buffer (2% SDS[Amresco], 10% glycerol [Thermo Fisher Scientific], 62.5 mM TRIS-HCL [pH6.8; Bio-Rad]) supplemented with protease inhibitor (Roche). Cell protein fractionation was performed with a subcellular protein fractionation kit for cultured cells (Thermo Fisher Scientific) according to the manufacturer’s instructions. For coimmunoprecipitation, HEK293T or C4-2B transiently transfected with the plasmids for 48 hours or C4-2B EnzR cells were lysed in Co-IP buffer (50mM tris-HCl pH 7.4 [Life Technology], 150mM NaCl [VWR], 1mM EDTA [Life Technology], 1% triton X-100 [Sigma-Aldrich]) supplemented with protease inhibitor (Roche). Whole lysates were incubated with primary antibodies overnight at 4°C with agitation, followed by 1 hour incubation with protein G-conjugated magnetic beads for mouse-derived Ab and protein A-conjugated for rabbit-derived Ab (SureBeads; Bio-Rad). Bound proteins were eluted with 1.5 × sample buffer for 10 minutes at 95°C with shaking at 1,050 rpm. The eluted protein complex was resolved in 10% SDS-PAGE gel and subjected to immunoblotting (see complete unedited blots in the supplemental material). As CXCR7 is a membrane protein, sample preparation caused its aggregation. Because protein aggregates are hard to dissolve, they generally accumulate at the border between stacking and resolving gels, obstructing their size resolution (68).

### Mass spectrometry and comprehensive phosphoproteome analysis.

Mass spectrometry analysis was done as reported previously (69). Briefly, LNCaP EnzR cells stably expressing GFP or CXCR7-FLAG were lysed in NETN buffer (100 mM NaCl [VWR], 20 mM Tris-Cl, pH 8.0 [Life Technology], 1 mM EDTA [Life Technology], and 0.5% NP-40 [Sigma-Aldrich]) containing protease inhibitor for 20 minutes at 4 °C. Crude lysates were subjected to centrifugation at 21,100*g* for 30 minutes. Supernatants were then incubated with anti-FLAG M2 agarose beads (Sigma-Aldrich) overnight at 4°C. The beads were washed 3 times with NETN buffer, and bounded proteins were eluted with 100 mM glycine HCL pH 2.5 (Thermo Fisher Scientific), which was then neutralized with 0.5M TRIS-HCL pH 8.0. The eluent was then subjected to mass spectrometry analysis. The enriched proteins in the CXCR7-FLAG sample are listed in Supplemental Table 3.

For comprehensive phosphoproteome analysis, LNCaP EnzR was transduced with pLKO or shCXCR7. Four days post-transduction, cells were lysed in 8 M Urea Lysis Buffer (8M Urea [Sigma-Aldrich], 50mM Tris, pH 8.2, 75mM NaCl) supplemented with protease inhibitor (Roche) and Halt Phosphatase Inhibitor Cocktail (Thermo Fisher Scientific) with sonication. Cell lysates were then processed through TiO2-based phosphopeptide enrichment followed by mass spectrometry analysis as described previously (34). The phosphopeptide enrichment data are presented in Supplemental Table 2. The changes in phospho-substrates were run through Kinase-Substrate Enrichment Analysis (KSEAapp) R package (70, 71).

### Microtubule binding assays.

To test microtubule binding, microtubules were preassembled at 37°C for 30 minutes from purified porcine-brain α/β-tubulin (Cytoskeleton Inc.) in PEM buffer (80 mm PIPES, pH 6.8 [Sigma-Aldrich], 1 mm MgCl2 [VWR], and 1 mm EGTA [Sigma-Aldrich]) supplemented with 50 μM taxol (Targetmol) and 1 mM GTP (Thermo Fisher Scientific). The binding assays were conducted by incubating 100 μg of protein lysates from 293T cells expressing CXCR7-Flag, incubated in lysis buffer (50mM tris-Cl pH7.5, 150mM NaCl, 1mM EDTA, 1% triton X-100 and 1mM MgCl2), with the preassembled microtubules at room temperature for 30 minutes. Once the incubation was competed, the samples were centrifuged at 100,000*g* for 15 minutes over 50% glycerol/PEM buffer. The resulting pellets and supernatants were analyzed by immunoblotting.

### GST pull-down assay.

To test protein-protein interaction in vitro, 1 μg of either GST-tagged ARRB2-full length, ARRB2-CTD, or GST-GFP (a negative control), were incubated for 2 hours at 4°C with 1 μg Flag-AURKA (Active Motif) in the binding buffer (25 mM tris-HCl, pH 7.5, 1% triton X-100, 150 mM NaCl, 1 mM MgCl2, 1 mM EDTA) supplemented with protease inhibitor (Roche). GST protein complex was separated by GSH-Sepharose (Thermo Fisher Scientific), eluted in 1 × SDS sample buffer, and subjected to immunoblotting.

### IF.

Cells were grown on poly D-lysine (Sigma-Aldrich) precoated coverslips and fixed with 4% paraformaldehyde for 15 minutes at room temperature, followed by permeabilization with 0.1% triton X-100 for 15 minutes at room temperature. After 3 washes with PBS, cells were incubated with a blocking buffer (5% BSA in PBS) for 30 minutes. Cells were incubated with primary antibody diluted in blocking buffer overnight at 4°C in a humidity chamber. The following antibodies were used: anti-CXCR7 (1:50; R&D, MAB42273), anti-clathrin heavy chain (1:50; CST, 4796), anti-GM130 (1:100; Proteintech, 11308-1-AP), anti-ARRB2 (1:100; Abclonal, A1171), anti-Aurora A (1:100; CST, 91590), and anti-α tubulin (1:100; Proteintech, 11224-1-AP). Next, slides were followed with secondary antibodies conjugated with Alexa Fluor 488 or Alexa Fluor 594 (A11034 or A11037, Invitrogen) for 1 hour. DAPI was used to counterstain nuclei. After 3 washes with PBS, coverslips were mounted on glass slides in Prolong Gold antifade reagent (Invitrogen). The cells were imaged by Nikon A1 Confocal Laser Microscope System. The images were analyzed by ImageJ (NIH).

### In situ PLA.

The detailed PLA procedure is described in supplemental materials. Briefly, cells were cultured on poly L-lysine (Sigma-Aldrich) precoated coverslips, fixed in 4% paraformaldehyde, and permeabilized in 0.1% triton X-100 solution in PBS. Coverslips were incubated with primary antibodies overnight and followed with Duolink in situ orange starter kit (Sigma-Aldrich) staining. The fluorescent images were taken on Nikon A1 confocal laser microscope system. PLA speckles were counted by ImageJ (NIH). The following antibodies were used: anti-CXCR7 (1:100; R&D, MAB42273), anti-AURKA (1:200; CST, 14475), anti-ARRB2 (1:200; Proteintech, 10171-1-AP), mouse IgG control (1:100; R&D, MAB002), and rabbit IgG control (1:200; Millipore,12-370).

### IHC.

IHC was performed on paraffin-embedded tissue sections. After deparaffinization, rehydration, and antigen retrieval with citrate buffer (Invitrogen), slides were permeabilized with 0.5% triton X-100. Slides were then blocked using a ready-to-use IHC kit (BioVision) as described by the manufacturer. Slides were then incubated with primary antibodies: anti-CXCR7 (1:50; R&D, MAB42273), mouse IgG control (1:100; R&D, MAB002), anti-phospho-Aurora A (Thr288) (1:1000; CST, 3079), mouse anti-SYP (1:500; Santa Cruz, sc-17750), and anti-AR (1:200; Santa Cruz, SC-186) overnight at 4°C in a humidity chamber. Slides were then washed with 1 × TBS 3 times for 5 minutes each time. For secondary antibodies, slides were incubated with IBSC-1-step HRP-anti-mouse, rat, and rabbit polymer provided in the kit. Slides were washed again with TBS (3 times for 5 minutes each time), then incubated with 3,3′- Diaminobenzidine (DAB) substrate for 1–5 minutes at room temperature. Slides were then counterstained with hematoxylin for 15 seconds, washed with running tap water, dehydrated in ethanol, cleared with xylene, and mounted with Permount (Fisher Chemical). Slides were visualized and imaged with an Olympus BX41 microscope bound to with Olympus UTV 0.5XC3 camera.

### Tissue microarray and PDX tumors.

Tissue microarrays (TMAs) of primary PCa (number of patients = 30, number of sites = 30) were generated at the Northwestern University Pathology Core through the prostate SPORE program. TMAs containing human CRPC and NEPC specimens were obtained from the University of Washington Medical Center Prostate Cancer Donor Program. All specimens were collected from patients within 8 hours of death, formalin-fixed (decalcified in formic acid for bone specimens), paraffin-embedded, and examined histologically for the presence of a nonnecrotic tumor. UWTMA79 was constructed with 1 mm–diameter core triplicates of visceral metastases and bone metastases (number of sites = 106) collected from 34 patients. UWTMA92 Array C was investigated, consisting of 1 mm–diameter core triplicates of visceral metastases and bone metastases (number of sites = 31) collected from 11 patients. Antibodies used in IHC include CXCR7 (1:100; R&D, MAB42273), AR (1:100; Biogenex, MU256-UC), and SYP (1:200; Santa Cruz, SC-17750). Images were captured with TissueFax Plus from TissueGnostics and exported to TissueFAX viewer. Immunostaining images were scored blindly by a pathologist. The intensity was scored as negative (score = 0), weak (score = 1), moderate (score = 2), or strong (score = 3), which was multiplied by the staining percentage to produce the product score for each core. LuCaP PDX tissue samples were provided by Eva Corey (University of Washington, Seattle, Washington, USA). LTL NEPC PDX tissue sections were supplied by Yuzhuo Wang (University of British Columbia, Vancouver, British Columbia, Canada).

### Xenograft models.

A total of 20 NSG male mice aged 5–8 weeks and bred at Northwestern University were inoculated with 1 × 10^6^ 22Rv1-GFP or 22Rv1-CXCR7 s.c. into the right dorsal flank in 50% solution of Cultrex UltiMatrix (R&D) in PBS. Once the tumors reached 100 mm^3^, tumor-bearing mice were randomized between 2 treatments, vehicle (5%DMSO, 30%PEG300, 5%Tween80) or alisertib (30 mg/kg/day), by oral gavage for 21 days once a day. Tumor size was measured twice a week and calculated by the formula (length (mm)×width^2^ (mm^2^) × 0.5). At the endpoint, mice were euthanized, and tumors were excised, fixed in 10% formalin, and subjected to paraffin embedding.

### Bioinformatics analysis.

RNA-Seq reads were mapped to NCBI human genome GRCh38 using STAR version 1.5.2. Raw counts of genes were calculated by STAR. Fragments Per Kilobase of transcript per Million mapped reads (FPKM) values were calculated by in-house Perl script. Differential gene expression was analyzed by the R Bioconductor DESeq2 package, which uses shrinkage estimation for dispersions. GSEA was performed following the manufacturer’s instructions (http://software.broadinstitute.org/gsea/index.jsp).

### Data availability.

The RNA-Seq data are available at public repository with GEO accession GSE199274. Mass spectrometry and comprehensive phosphoproteome data are provided as supplemental materials (Supplemental Tables 2 and 3). Data analyses were performed using the packages available at Bioconductor or CRAN using default parameters.

### Statistics.

A 2-tailed unpaired Student’s *t* test was used to evaluate data consisting of 2 groups. A 1-way ANOVA paired with Dunnett’s multiple comparison tests was used to evaluate data consisting of 3 and more groups. A 2-way ANOVA paired with Bonferroni correction was used to determine statistical significance in experiments comparing 2 repeatedly measured groups. A 2-way ANOVA paired with Tukey correction was used to determine statistical significance in experiments comparing 3 and more repeatedly measured groups. *P* < 0.05 indicates statistical significance. GraphPad Prism 9 was used for statistical analysis.

### Study approval.

The Northwestern University IACUC (Chicago, Illinois, USA) approved all animal studies. TMAs of primary PCa generated at the Northwestern University Pathology Core were approved by Northwestern University IRB. UWTMA79 and UWTMA92 were approved and provided by the University of Washington Medical Center through the Prostate Cancer Donor Rapid Autopsy Program, which is approved by the University of Washington IRB.

## Author contributions

GG and JY conceived the project and designed experiments. KF performed tubulin binding and GST pull–down assays, XL performed RNA-seq and cultured NCI-H660, ZL performed the cell proliferation assay, WX contributed to the animal study, SA performed PLA, and GG performed the rest of the experiments. JCZ, GG, and JY conducted the bioinformatic and statistical analysis. GES consulted on CXCR7 targeting, HB on the Alisertib trial, and MH on AURKA inhibitors. CM provided TMA data. EC provided LuCaP PDX tissue blocks. YW and DL provided LTL NEPC PDX tissue sections. JY and GG designed the figures and wrote the manuscript. All authors read and commented on the manuscript.

## Supplementary Material

Supplemental data

Supplemental table 2

Supplemental table 3

Supporting data values

## Figures and Tables

**Table 1:**
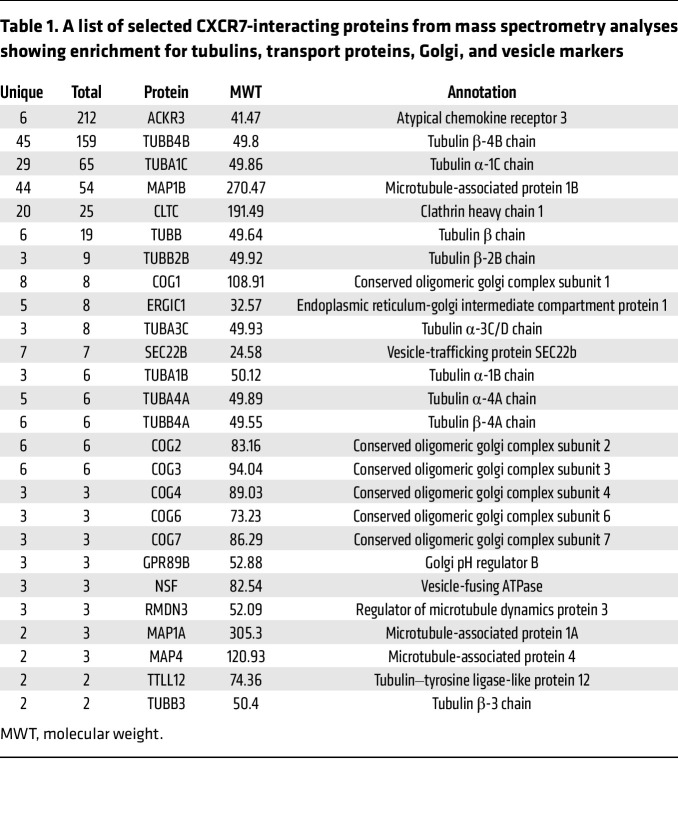
A list of selected CXCR7-interacting proteins from mass spectrometry analyses showing enrichment for tubulins, transport proteins, Golgi, and vesicle markers

## Version 1 06/22/2023

In-Press Preview

## Version 2 08/01/2023

Electronic publication
