# Supplementary material for: Chemokine receptor CXCR7 activates Aurora Kinase A and promotes neuroendocrine prostate cancer growth
Source: J Clin Invest. 2023 Aug 1;133(15):e166248. doi: 10.1172/JCI166248 (PMC10378179; doi:10.1172/JCI166248)

Supplementary Materials

Chemokine receptor CXCR7 activates AURKA and promotes neuroendocrine prostate cancer growth

Galina Gritsina[1], Ka-wing Fong[1,2], Xiaodong Lu[1,3], Zhuoyuan Lin[1,4], Wanqing Xie[1,3], Shivani Agarwal[1], Dong Lin[5,6], Gary E. Schiltz[7,8], Himisha Beltran[9], Eva Corey[10], Colm Morrissey[10], Yuzhuo Wang[5,6], Jonathan C. Zhao[1,3,7,12,13], Maha Hussain[1,7], Jindan Yu[1,3,7,11,12,13]

[1]Division of Hematology/Oncology, Department of Medicine, Northwestern University Feinberg School of Medicine, Chicago, IL, USA.
[2]Department of Toxicology and Cancer Biology, University of Kentucky, Lexington, KY, USA
[3]Department of Urology, Emory University School of Medicine, Atlanta, GA, USA.
[4]Department of Urology, the Second Affiliated Hospital of Guangzhou Medical University, Guangzhou, China.
[5]Department of Experimental Therapeutics, BC Cancer Agency, Vancouver, BC, V5Z1L3, Canada
[6]Vancouver Prostate Centre, Department of Urologic Sciences, University of British Columbia, Vancouver, BC, V6H 3Z6 Canada
[7]Robert H. Lurie Comprehensive Cancer Center, Northwestern University Feinberg School of Medicine, Chicago, IL, USA.
[8]Department of Chemistry, Northwestern University, Evanston, IL, USA.
[9]Department of Medical Oncology, Dana Farber Cancer Institute, Boston, Massachusetts, USA.
[10]Department of Urology, University of Washington, Seattle, WA, USA.
[11]Department of Biochemistry and Molecular Genetics, Northwestern University, Chicago, IL, USA
[12]Department of Human Genetics, Emory University School of Medicine, Atlanta, GA, USA.
[13]Winship Cancer Institute, Emory University School of Medicine, Atlanta, GA, USA

Supplementary Methods

Supplementary Reference

Supplementary Figures S1-S7

Figure S1. CXCR7 is up-regulated in neuroendocrine prostate cancer

Figure S2. CXCR7 promotes mitotic spindle and cell cycle processes

Figure S3. CXCR7 promotes AURKA signal transduction pathway

Figure S4. CXCR7-ARRB2 protein complex interacts with AURKA

Figure S5. CXCR7 is transported along the microtubules to the Golgi apparatus

Figure S6. CXCR7 is transported along the microtubules to the pericentrosomal Golgi apparatus

Figure S7. CXCR7 increases PCa growth, which is abolished by AURKA inhibition

Supplementary Table 1. Oligonucleotide sequences used in the study

Supplementary Methods

Cell Culture and Reagents

C4-2B, LNCaP, 22Rv1, NCI-H660, and HEK293T cell lines were obtained from the ATCC. LNCaP, C4-2B, 22Rv1 cells were cultured in RPMI1640 with 10% fetal bovine serum (FBS) and 1% penn/strep. HEK293T cells were cultured in DMEM with 10% FBS. Enz-resistant LNCaP and C4-2B have been described previously (1) and were cultured in RPMI1640 with 10% FBS, 1% penn/strep supplemented with 1 μM or 10 μM enzalutamide respectively. NCI-H660 was cultured in RPMI supplemented with 5% FBS, 0.005mg.ml insulin, 0.01mg/ml transferrin, 30nM sodium selenite, 10nM hydrocortisone, 10nM beta-estradiol, 4 mM L-glutamine. The cell lines were authenticated (Genetica DNA Laboratories) and routinely confirmed to be free from mycoplasma by PCR test (2). Primers are listed in **Supplementary Table 1.** For CXCR7 stimulation experiments, C4-2B cells were starved in serum-free culture media for 48 hours and treated with 100 ng/ml of SDF1 (R&D). Enzalutamide (Enz) (S1250) was purchased from Selleckchem, both nocodazole (T2802) and alisertib (T2241) were purchased from TargetMol.

Western blotting, co-immunoprecipitation, protein fractionation

Proteins (20-40 μg) were resolved in 10% SDS-PAGE gel and then transferred onto PVDF membranes (Millipore). After blocking the membranes with 5% fat-free milk to total protein or 3% BSA for phospho-protein in TBST for 1 hour at room temperature, the membranes were incubated with appropriate dilutions of specific primary antibodies in blocking buffer overnight at 4°C. After washing, the blots were incubated with HRP-conjugated secondary antibodies (Jackson Labs Antibodies) for 1h and visualized using the ECL substrate (Amersham) on the Chemidoc imaging system (Bio-Rad).

The following antibodies were used: anti-pAurora A(Thr288)/B(Thr232)/C(Thr198) (CST, 2914), anti-pTACC3 (Ser558) (CST, 8842), anti-TACC3 (CST, 8069), anti-Aurora A (CST, 91590), anti-GM130 (Proteintech, 11308-1-AP), anti-ARRB2 (Abclonal, A1171), anti-EGFR (Bethyl, A300-388A-T), anti-HDAC3 (Santa Cruz, sc-376957), anti-KRT8/18 (CST, 4546), anti-β-Actin (Santa Cruz, sc-47778), anti-GAPDH (CST, 2118), anti-α tubulin (Proteintech, 11224-1-AP), anti-γ tubulin (Proteintech, 15176-1-AP), anti-HA (CST, 3724), anti-Myc (CST, 2278), anti-FLAG (Sigma, F7425), antibodies used for immunoprecipitation: anti-HA (Santa cruz, sc-7392), anti-FLAG M2(Sigma, F1804), anti-CXCR7 (Proteintech, 20423-1-AP).

Cell proliferation and cell viability assays

Cell proliferation was measured with WST-1 (Promega) as described by the manufacturer. In brief, 3000-5000 cells seeded in 96-well plates were cultured for up to 6 days. The cells were incubated with WST-1 for 1 hour at 37°C and 5% CO2. The absorbance was measured at 440 nm using the KC4 microplate reader (BioTek) and blanked to the no-cell media control. Statistical analysis of normalized WST1 readings was performed by two-way analysis of variance (ANOVA) with either Tukey, or Bonferroni post-hoc test at the significance level of alpha equals 0.05 by Prism 9 (GraphPad Software).

Fluorescence-activated flow cytometry

The fluorescence-activated flow cytometry cells were fixed and permeabilized with 1x Fix/Perm buffer (eBioscience). Cells were stained with primary antibodies for 1 hour at 4°C and followed with secondary anti-mouse antibodies conjugated with Alexa 594 (Invitrogen). Data were obtained on LSRII (BD), and analysis was performed with FlowJo (BD).

In situ Proximity ligation assay (PLA)

To determine interaction between endogenous CXCR7 and AURKA or CXCR7 and ARRB2 we used Duolink® in Situ Orange Starter Kit for Mouse/Rabbit antibody combination (Sigma Aldrich, DUO92102) according to the manufacturer's instructions. C4-2B cells were growrn on poly-D-lysine-coated coverslips overnight and fixed with 4% paraformaldehyde for 15 min at room temperature, followed by permeabilization with 0.1% Triton X-100 for 15 min at room temperature. After 3 washes with PBS, cells were incubated with supplied blocking buffer for 30 min. Then cells were incubated with primary antibody diluted in blocking buffer overnight at 4°C in a humidity chamber. The following antibodies were used: anti-CXCR7 (1:100, MAB42273, R&D), anti-AURKA (1:200, CST 14475), anti-ARRB2 (1:200, Proteintech 10171-1-AP), or mouse IgG control (1:100, MAB002, R&D), rabbit IgG control (1:200, 12-370, Millipore). The next day slips were incubated with the secondary antibodies conjugated with complementary oligonucleotide PLA probes: anti-rabbit PLUS and anti-mouse MINUS, at 37°C for 1 h. The ligation, amplification and washing steps were performed as instructed. The coverslips were stained with DAPI, mounted, and subjected for confocal microscopy by Nikon A1 Confocal Laser Microscope System. PLA speckles were counted with ImageJ (NIH).

In order to evaluate the effect of ARRB2 on CXCR7 interaction with AURKA, C4-2B cells seeded in pre-coated slides were transfected with 2.5ug pCDNA3 or pCDNA3-Venus-ARRB2 constructs for 48h then subjected to PLA protocol. For the study of the effect from acute nocodazole treatment, pre-seeded C4-2B were treated with 10ug/ml nocodazole or DMSO for 1h then subjected to PLA protocol. The number of speckles from 3-5 images was normalized by the

number of nuclei. Statistical analysis was performed by unpaired t-test by Prism 9 (GraphPad Software).

References:

1. Li S, Fong KW, Gritsina G, Zhang A, Zhao JC, Kim J, *et al.* Activation of MAPK Signaling by CXCR7 Leads to Enzalutamide Resistance in Prostate Cancer. Cancer Res **2019**;79:2580-92
2. Uphoff CC, Drexler HG. Detecting mycoplasma contamination in cell cultures by polymerase chain reaction. Methods Mol Biol **2011**;731:93-103

Supplementary Figures

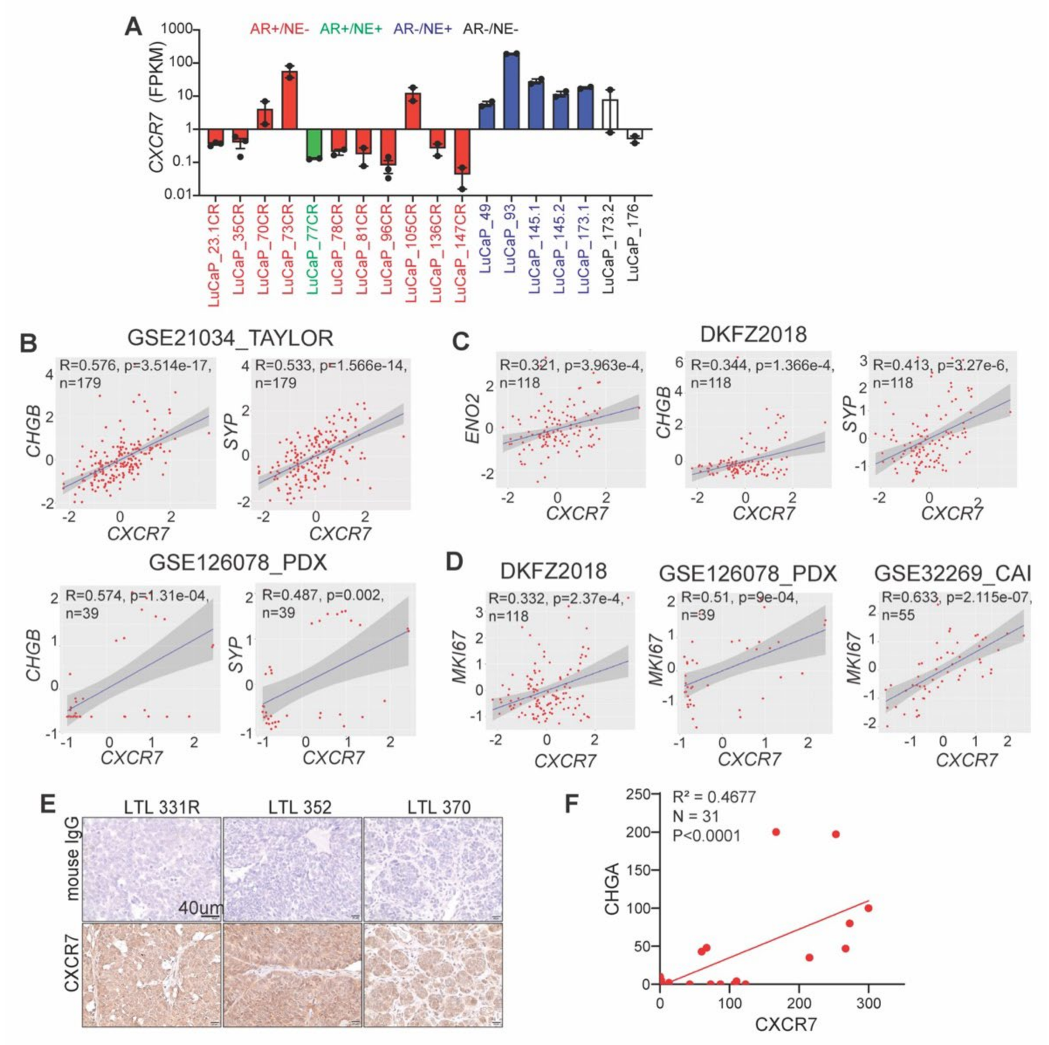

Figure S1. CXCR7 is up-regulated in neuroendocrine prostate cancer

(A) Histogram shows *CXCR7* mRNA expression across a panel of LuCaP PDX models. Color coding represents LuCaP PDX phenotype. **(B)** Scatter plots show a significant correlation between *CXCR7* and *CHGB*, or *SYP* in the PCa patient datasets. The dark grey area indicates the 95% confidence interval, x- and y-axes show normalized expression. **(C)** Scatter plots show correlation between *CXCR7* and *ENO2*, *CHGB*, or *SYP* in the advance PCa dataset. The dark grey area indicates the 95% confidence interval, x- and y-axes show normalized expression. **(D)** Scatter plots show a significant correlation between *CXCR7* and in multiple PCa patient datasets. The dark grey area indicates the 95% confidence interval, x- and y-axes show normalized expression. **(E)** Representative IHC staining of CXCR7 or mouse IgG isotype control in the selected NEPC LTL PDX tumors. **(F)** Correlation between CXCR7 and CHGA IHC staining scores in TMAs. Every dot represents the average intensity score of three cores for each tumor. A total of 31 tumors were analyzed. Statistical analysis is based on simple linear regression.

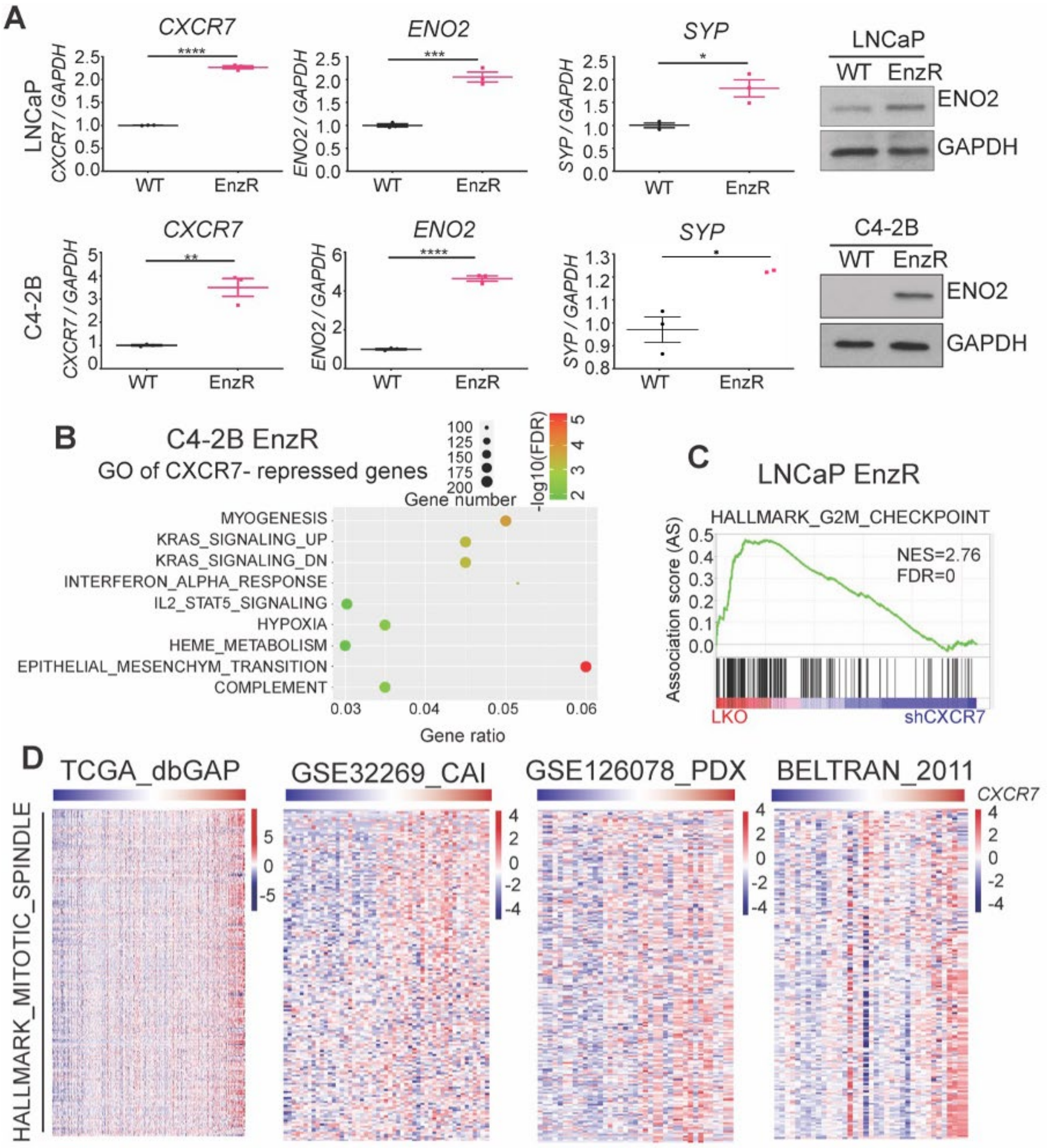

Figure S2. CXCR7 promotes mitotic spindle and cell cycle processes

(A) *CXCR7* and NEPC marker genes are up-regulated in LNCaP-EnzR and C4-2B-EnzR cells compared to parental (WT) controls. QRT-PCR and WB were used to show increased expression of *CXCR7, ENO2, SYP*. QRT-PCR data were normalized to *GAPDH* and then the control condition (mean ± SEM, n=3), Significant difference between control vs KD cells was calculated by two-tailed unpaired Student's t-test, (**P*<0.05, ** *P* <0.01, *** *P* <0.001, **** *P* <0.0001). **(B)** Gene ontology analysis show signaling categories that were up-regulated upon *CXCR7* knockdown (KD) in C4-2B-EnzR cells. **(C)** Gene Set Enrichment Analysis (GSEA) showing enrichment of HALLMARK_G2M_CHECKPOINT molecular signature in *CXCR7*-KD LNCaP-EnzR cells relative to control (LKO). NES: normalized enrichment score. FDR: false discovery rate. **(D)** Heatmap view of the HALLMARK_MITOTIC_SPINDLE molecular signature genes in the indicated prostate cancer patient databases with samples ordered by *CXCR7* level (top row).

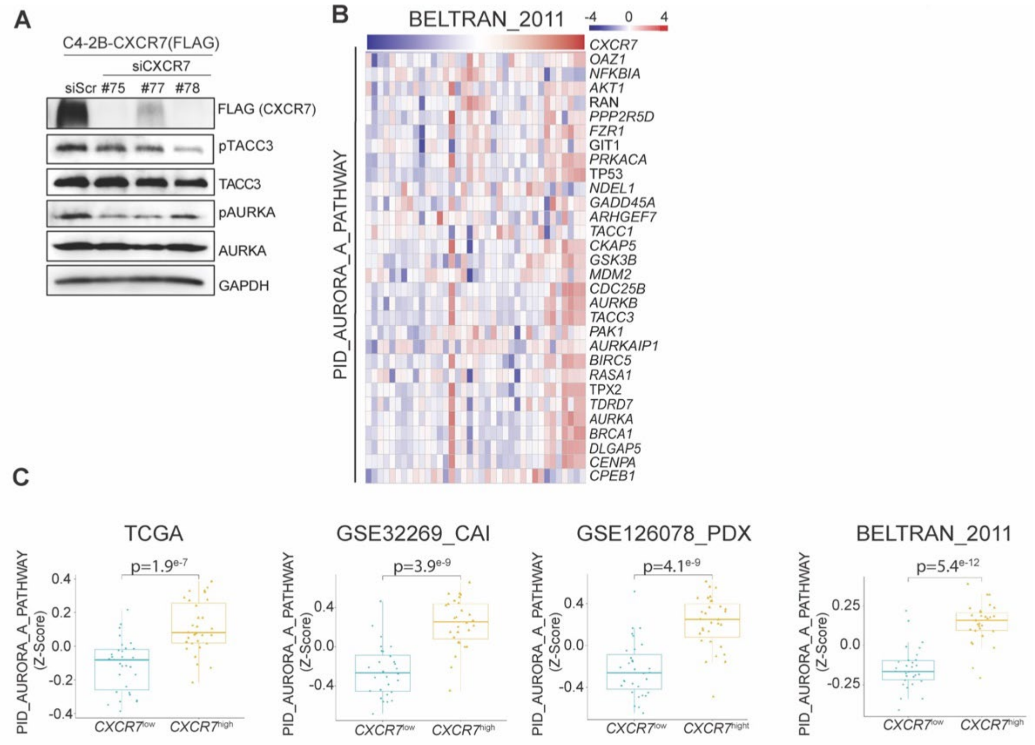

Figure S3. CXCR7 promotes AURKA signal transduction pathway

(A) CXCR7 overexpressing C4-2B cells were treated with siCXCR7 for 48h. The protein lysates were resolved and probed for the designated markers.

(B) Heatmap view of the PID_AURORA_A_PATHWAY molecular signature genes in the indicated prostate cancer patient dataset ordered by *CXCR7* expression level.

(C) Z-score plots show a significant difference in AURKA signature gene expression in *CXCR7*-high versus *CXCR7*-low samples. Samples in each data set were divided into *CXCR7*-low vs -*CXCR7*-high groups in the middle. Z-scores were calculated as (expression value-mean)/(standard deviation across each group of samples). Value is the log2(gene expression of RNA-seq+1) of each sample. Statistical test is based on two-tailed unpaired Student's t-test.

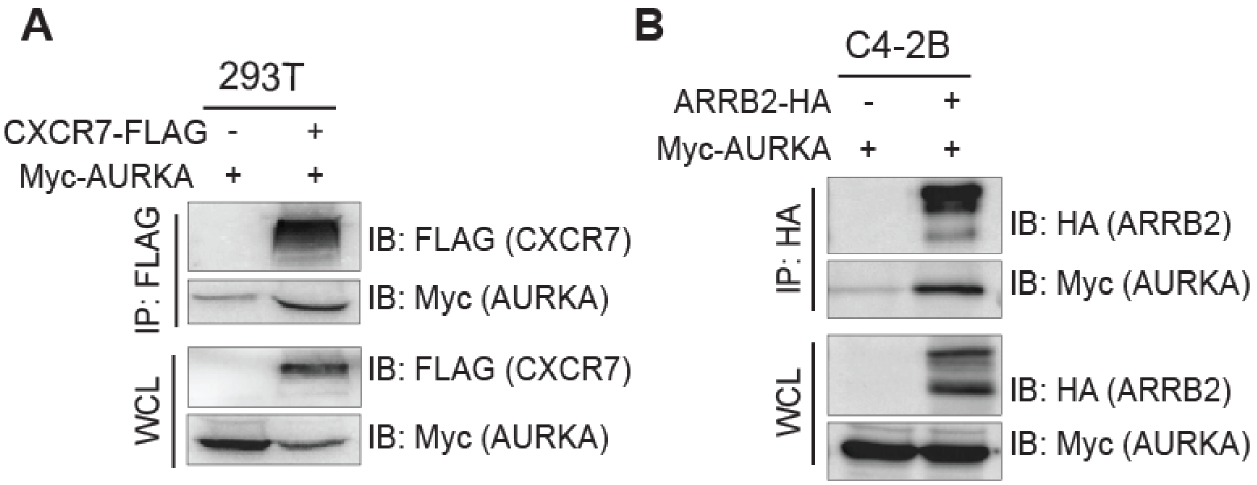

Figure S4. CXCR7-ARRB2 protein complex interacts with AURKA
(A) 293T cells were co-transfected with Myc-AURKA and CXCR7-FLAG or empty vector control for 48 hours and subjected to co-immunoprecipitation (co-IP) with anti-FLAG antibodies and probed for Myc and FLAG. **(B)** C4-2B cells were transiently co-transfected with ARRB2-HA (hemagglutinin) and Myc-AURKA for 48h and subjected to co-IP with anti-HA antibodies. The eluted protein complex was resolved and probed for Myc and HA.

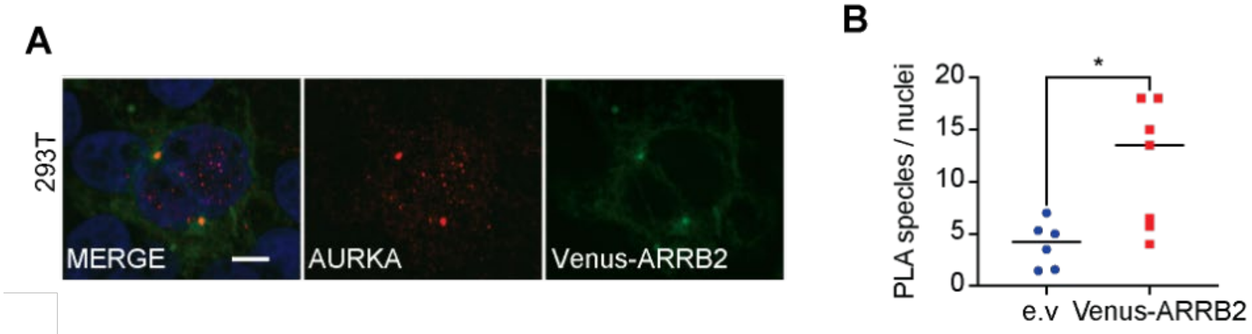

Figure S5. CXCR7 is transported along the microtubules to the Golgi apparatus
(A) Centrosomal AURKA co-localizes with ARRB2. 293T were transfected with Venus-ARRB2 (green fluorescence) and stained for endogenous AURKA (red). Image scale bar 5 µm.
(B) The number of speckles was counted and normalized to the number of nuclei per imaged field view. The experiment was performed once. (Number of field views: n=6 (empty vector control), n=7 (Venus-ARRB2), two-tailed unpaired Student's t-test, **P*<0.05).

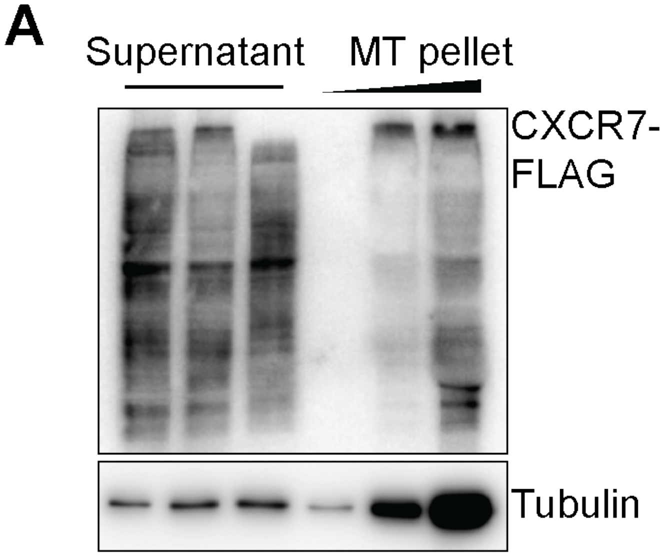

Figure S6. CXCR7 is transported along the microtubules to the pericentrosomal Golgi apparatus

(A) In vitro microtubule binding assay shows tubulin-dependent CXCR7-FLAG accumulation in the microtubule pellet. Increasing amount of pre-assembled microtubules were incubated with CXCR7-FLAG-expressing 293T cell lysate for 30 min at room temperature, then submitted to ultracentrifugation, and resolved by WB.

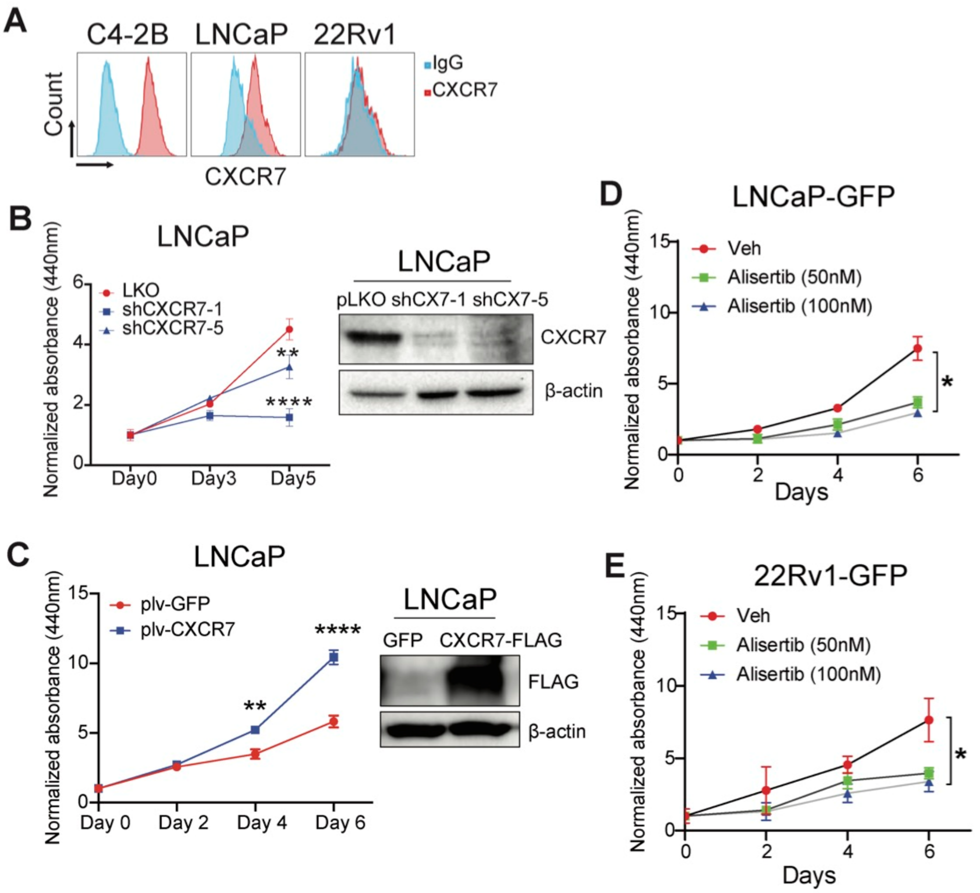

Figure S7. CXCR7 increases PCa growth, which is abolished by AURKA inhibition

(A) Flow cytometry charts show the expression level of CXCR7 across three PCa cell lines.

(B) *CXCR7* knocking down (KD) reduces LNCaP cell proliferation. WST1 assay was performed to measure cell proliferation. *CXCR7* KD was confirmed by WB. Representative proliferation data from three repeated experiments are shown here. The data were analyzed by two-way ANOVA followed by Tukey corrected multiple-comparison test (mean±SD, n=3, ***P*<0.01, *****P*<0.0001).

(**C**) CXCR7 overexpression increases LNCaP cell proliferation. Overexpression was confirmed by WB. Representative proliferation data from two repeated experiments are shown here. The data were analyzed by two-way ANOVA followed by Bonferroni-corrected multiple-comparison test (mean±SD, n=3, ***P*<0.01, *****P*<0.0001).

(D-E) Alisertib treatment decreases cell proliferation in control LNCaP-GFP **(D)** and 22Rv1-GFP **(E)** but to the less extent than in LNCaP-CXCR7 and 22Rv1-CXCR7 (**Figure 7C-D)**. Representative data from one **(D)** and two **(E)** experiments are shown. The data were analyzed by two-way ANOVA followed by Tukey-corrected multiple-comparison test (mean±SD, n=3, **P*<0.05).

Supplementary Table 1. Oligonucleotide sequences used in the study

Name	Sequence	Application
FW Myco-5-1	CGCCTGAGTAGTACGTTCGC	Mycoplasma testing
FW Myco-5-2	CGCCTGAGTAGTACGTACGC	Mycoplasma testing
FW Myco-5-3	TGCCTGAGTAGTACATTCGC	Mycoplasma testing
FW Myco-5-4	TGCCTGGGTAGTACATTCGC	Mycoplasma testing
FW Myco-5-5	CGCCTGGGTAGTACATTCGC	Mycoplasma testing
FW Myco-5-6	CGCCTGAGTAGTATGCTCGC	Mycoplasma testing
RV Myco-3-1	GCGGTGTGTACAAGACCCGA	Mycoplasma testing
RV Myco-3-2	GCGGTGTGTACAAAACCCGA	Mycoplasma testing
RV Myco-3-3	GCGGTGTGTACAAACCCCGA	Mycoplasma testing
CCND1 F1	CCCTCGGTGTCCTACTTCAA	qRT-PCR
CCND1 R1	AGGAAGCGGTCCAGGTAGTT	qRT-PCR
E2F1 F4	CAGCTGGACCACCTGATGAAT	qRT-PCR
E2F1 R4	GCAATGCTACGAAGGTCCTGA	qRT-PCR
CDK1 F	GGAAACCAGGAAGCCTAGCATC	qRT-PCR
CDK1 R	GGATGATTCAGTGCCATTTTGCC	qRT-PCR
BIRC5 F	CAAGGACCACCGCATCTCTAC	qRT-PCR
BIRC5 R	AGTCTGGCTCGTTCTCAGTGG	qRT-PCR
TPX2 F	TTCAAGGCTCGTCCAAACACCG	qRT-PCR
TPX2 R	GCTCTCTTCTCAGTAGCCAGCT	qRT-PCR
AURKA F	AAGGACACAAGACCCGCTG	qRT-PCR
AURKA R	CCTGTTAAGGCTACAGCTCCA	qRT-PCR
CXCR7 F2	AGCAGCAGGAAGAAGATGGT	qRT-PCR
CXCR7 R2	CGTGACGGTCTTCAGGTAGT	qRT-PCR
GAPDH F1	TGCACCACCAACTGCTTAGC	qRT-PCR
GAPDH R1	GGCATGGACTGTGGTCATGAG	qRT-PCR
CXCR7-FLAG FW	CGATGGATCTGCATCTCTTCGACTACTCAG	cloning
CXCR7-FLAG REV	TCACTTATCGTCGTCATCCTTGTAATCTTT GGTGCTCTGCTCCAAGGCA	cloning

ARRB2-Cd Forward	GCACCATGGGCCCCCAGCCATCAGCTGAAACC	ARRB2 C-domain cloning
ARRB2-Cd Reverse	GGGGGCCCATGGTGCGCGTGGTACCA	ARRB2 C-domain cloning
ARRB2-Nd Forward	AGACACCCTGCTACCCATACGACGTCCC	ARRB2 N-domain cloning
ARRB2-Nd Reverse	GGTAGCAGGGTGTCTCAGGAGCAAACTG	ARRB2 N-domain cloning
AuroA-RegDom_FW	TGGAAGACTACCCAACTTTCTTGTAC AAAGTGG	Cloning Reg Dom of Aurora A
AuroA-RegDom_RV	TTGGGTAGTCTTCCAAAGCCCACTGCC	Cloning Reg Dom of Aurora A
AuroA-KinDom_FW	AAGTTGGCTTTGAAATTGGTCGCCCTCTG	Cloning Kin Dom of Aurora A
AuroA-KinDom_RV	TTTCAAAGCCAACTTTTTTGTACAAACTTGT	Cloning Kin Dom of Aurora A
GST-ARRB2-FL-FW	ATCCCCGGAATTCCCAATGGGTG AAAAACCCGGGACC	ARRB2 full-length cloning for GST pull-down
GST-ARRB2-FL-RV	CGCTCGAGTCGACCCGCAGAACTG GTCATCACAGTCG	ARRB2 full-length cloning for GST pull-down
GST-ARRB2-Cdom-FW	ATCCCCGGAATTCCCAATGGGCCC CCAGCCATC	ARRB2 C-domain cloning for GST pull-down
GST-ARRB2-Cdom-RV	CGCTCGAGTCGACCCGCAGAACTGG TCATCACAGTCG	ARRB2 C-domain cloning for GST pull-down

Uncropped images of immunoblots relative to Figure S2A. Black boxes indicate where images were cropped.

LNCAP

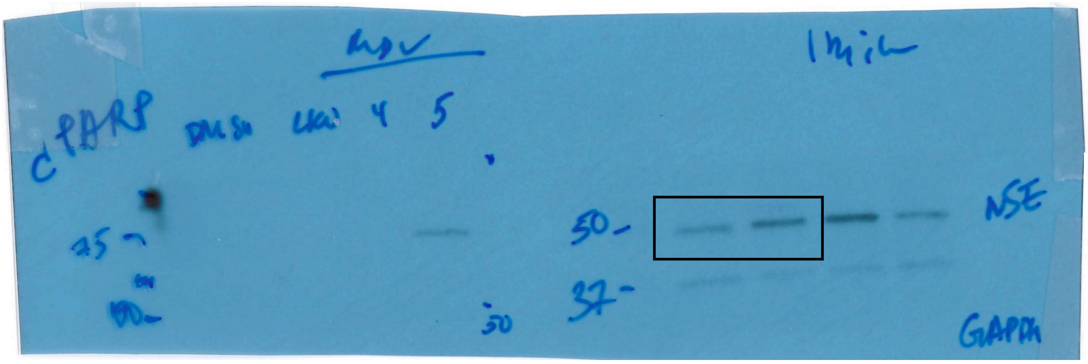

ENO2

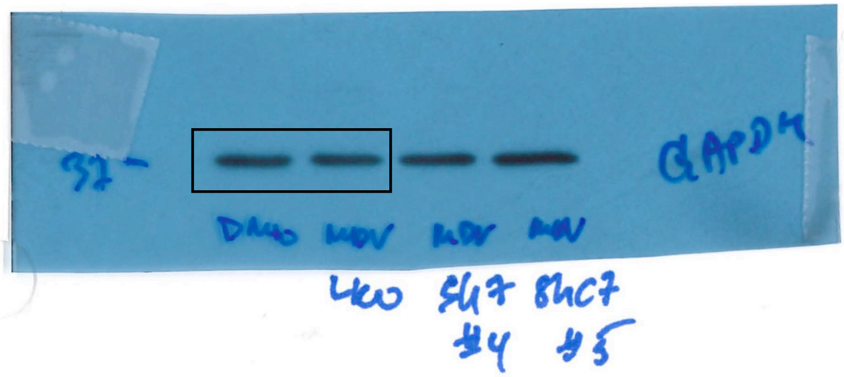

GAPDH

C4-2B

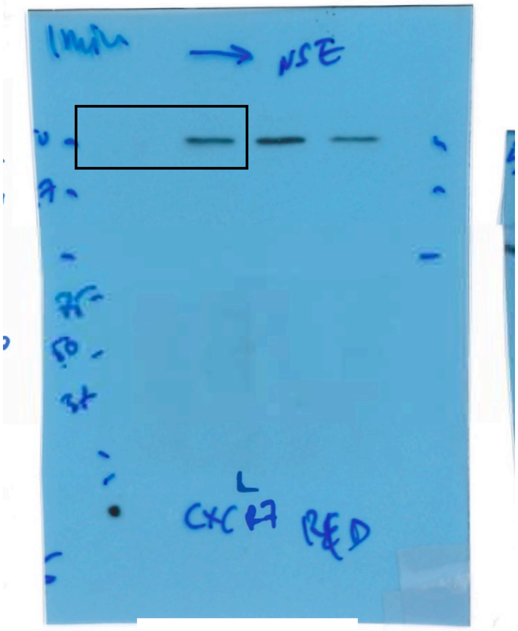

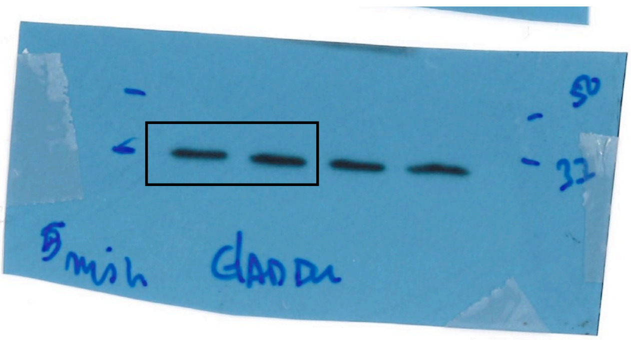

ENO2

Uncropped images of immunoblots relative to Figure 3D. Black boxes indicate where images were cropped.

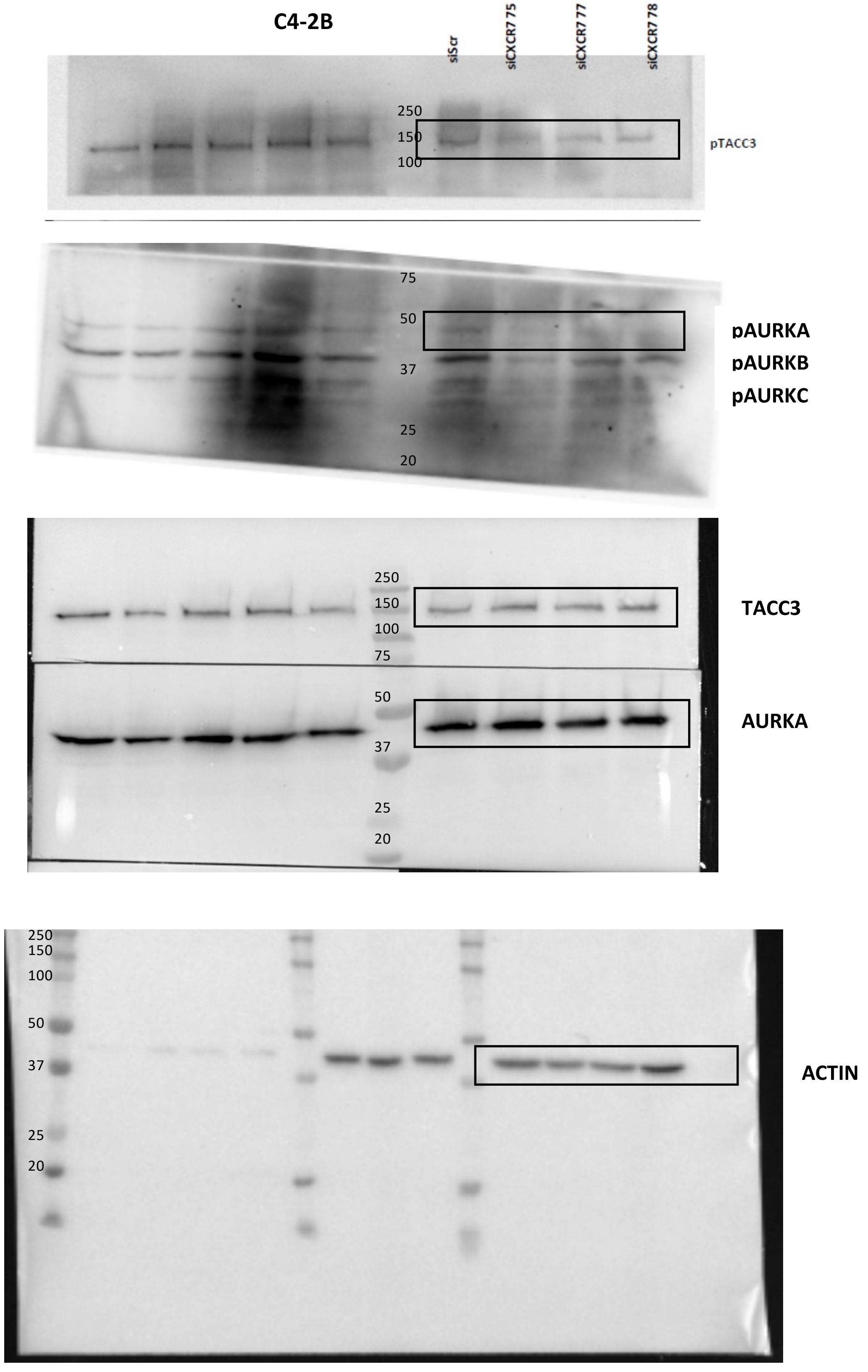

Uncropped images of immunoblots relative to Figure 3E. Black boxes indicate where images were cropped.

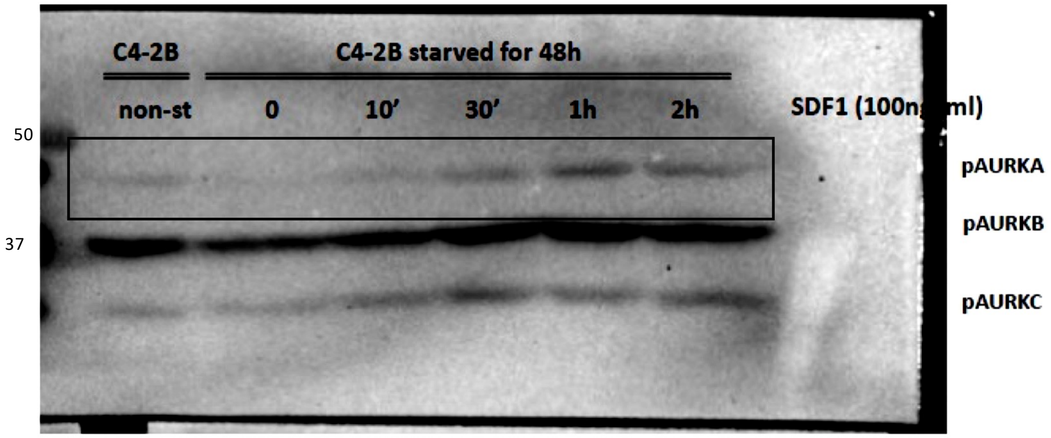

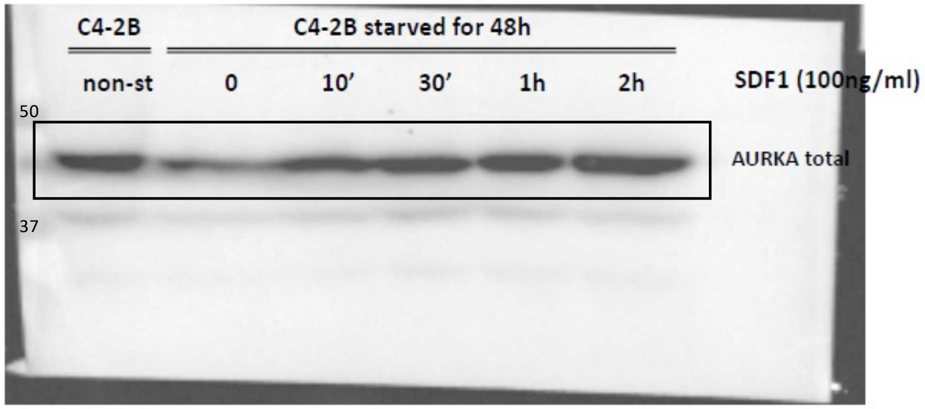

Uncropped images of immunoblots relative to Figure 3G. Black boxes indicate where images were cropped.

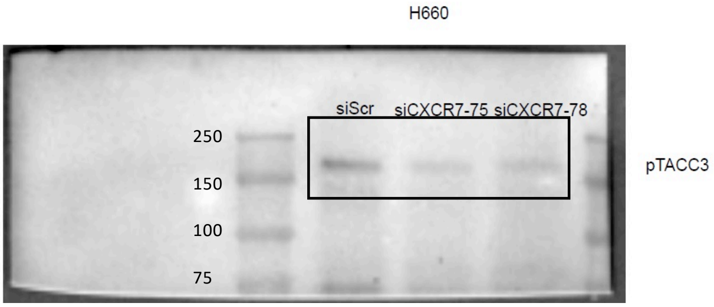

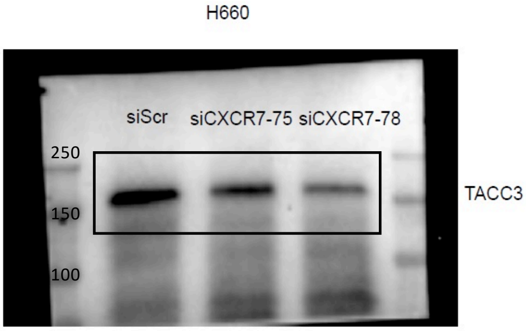

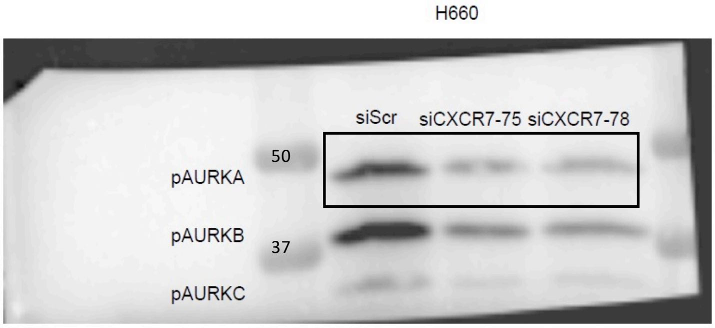

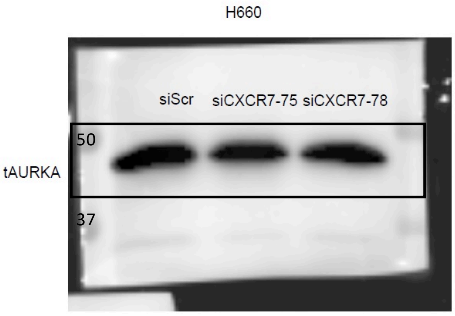

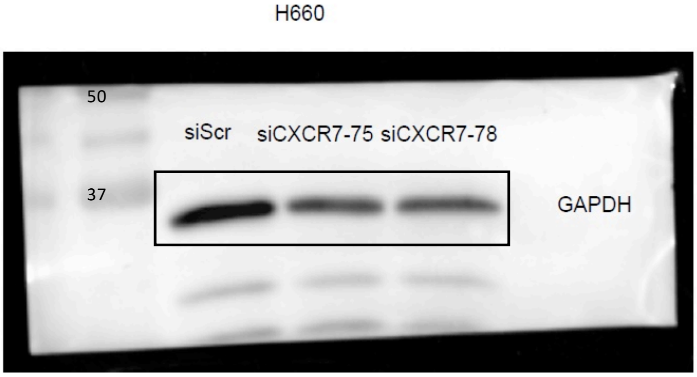

Uncropped images of immunoblots relative to Figure S3A. Black boxes indicate where images were cropped.

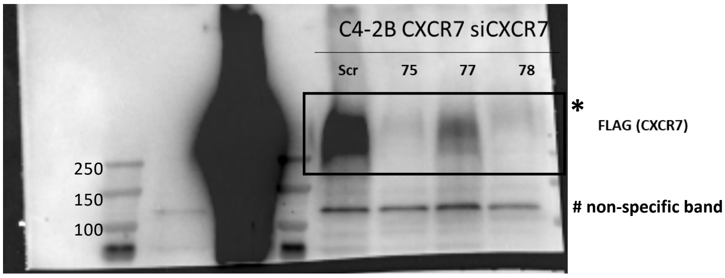

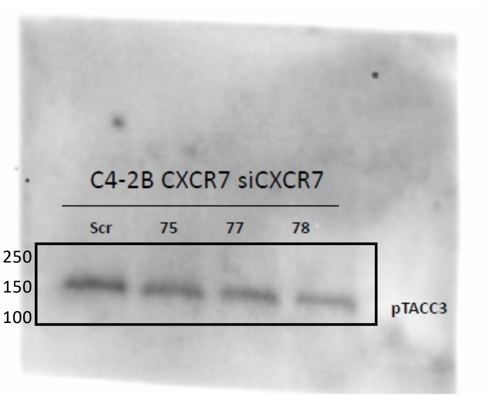

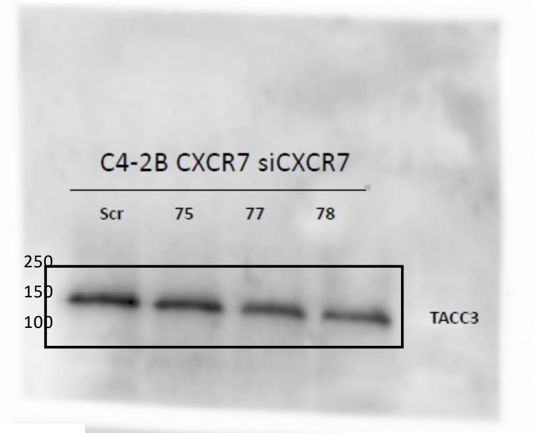

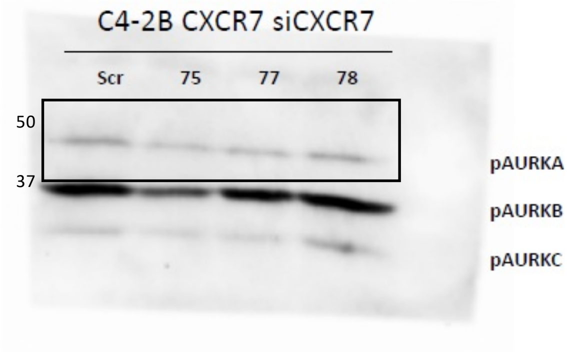

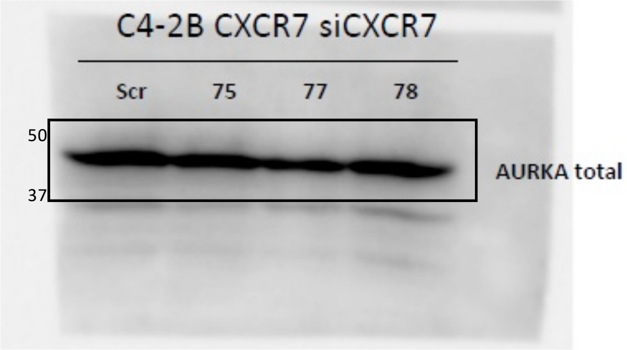

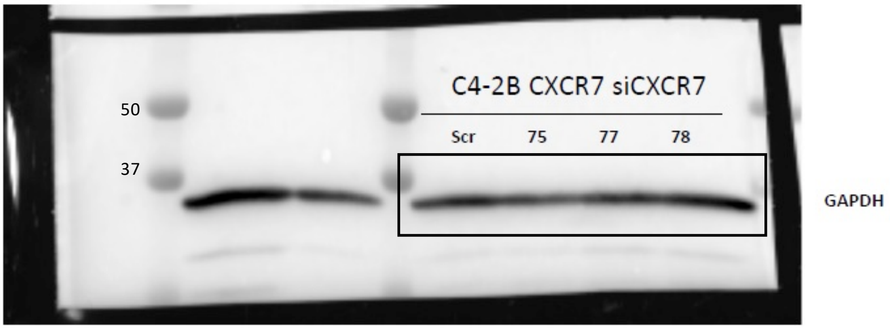

*CXCR7 becomes aggregated during IP preparation and accumulates at the border between stacking/resolving gels

Uncropped images of immunoblots relative to Figure 4. Black boxes indicate where images were cropped.

A

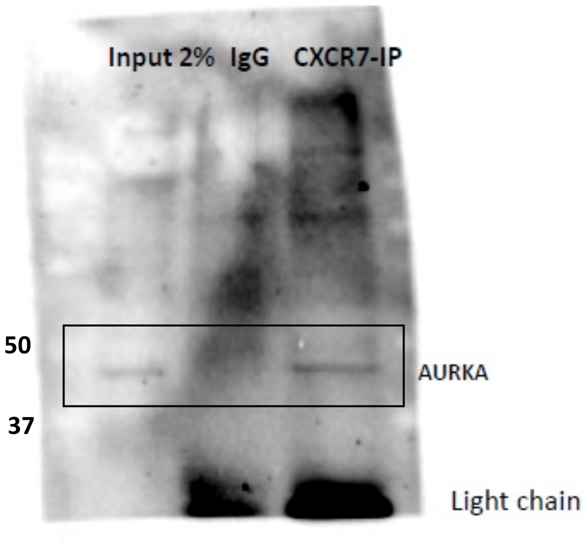

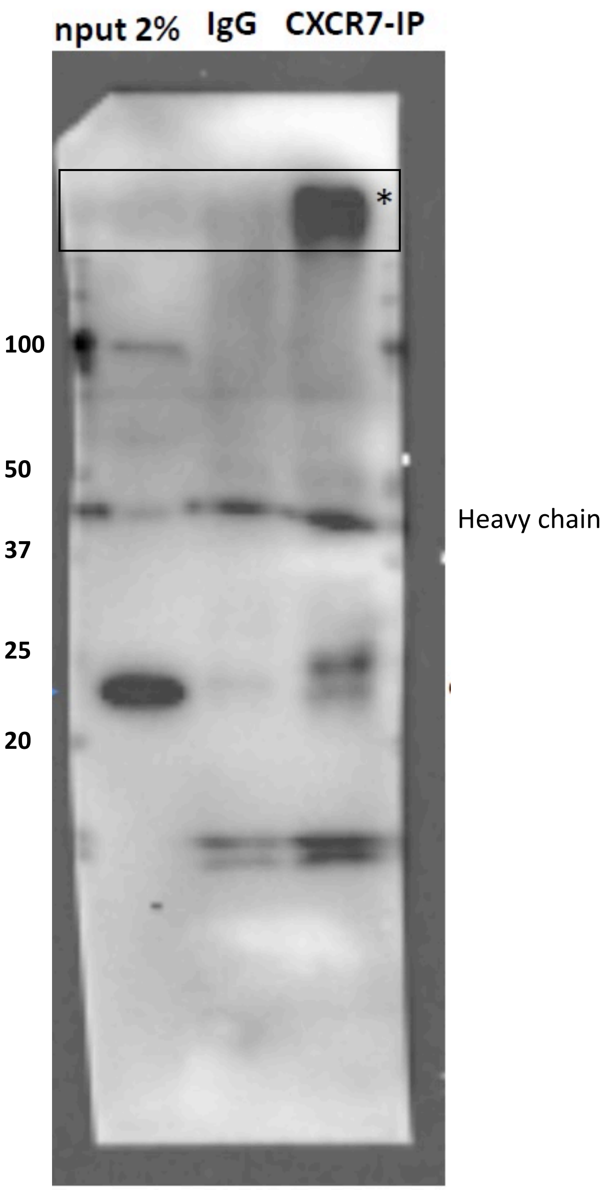

*CXCR7 becomes aggregated during IP preparation and accumulates at the border between stacking/resolving gels

Uncropped images of immunoblots relative to Figure 4. Black boxes indicate where images were cropped.

B

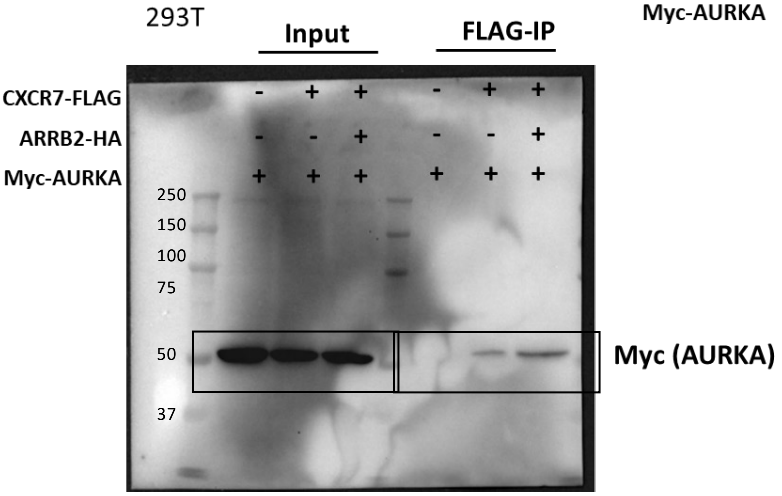

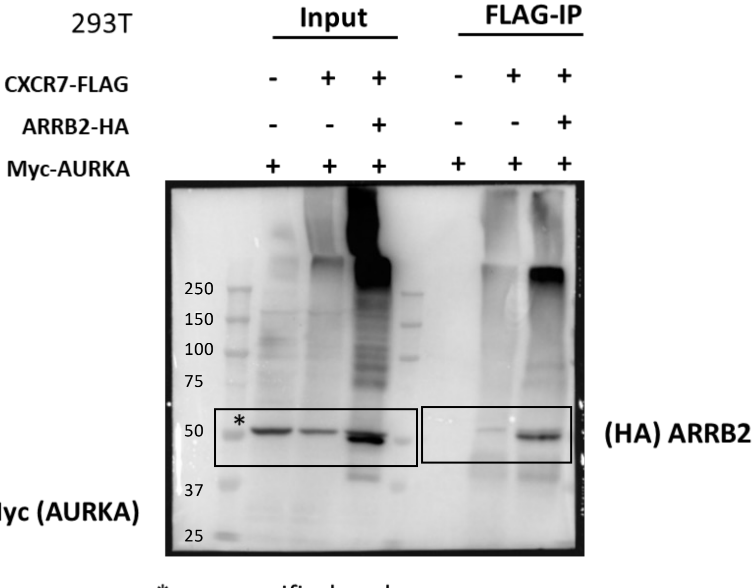

*non-specific band

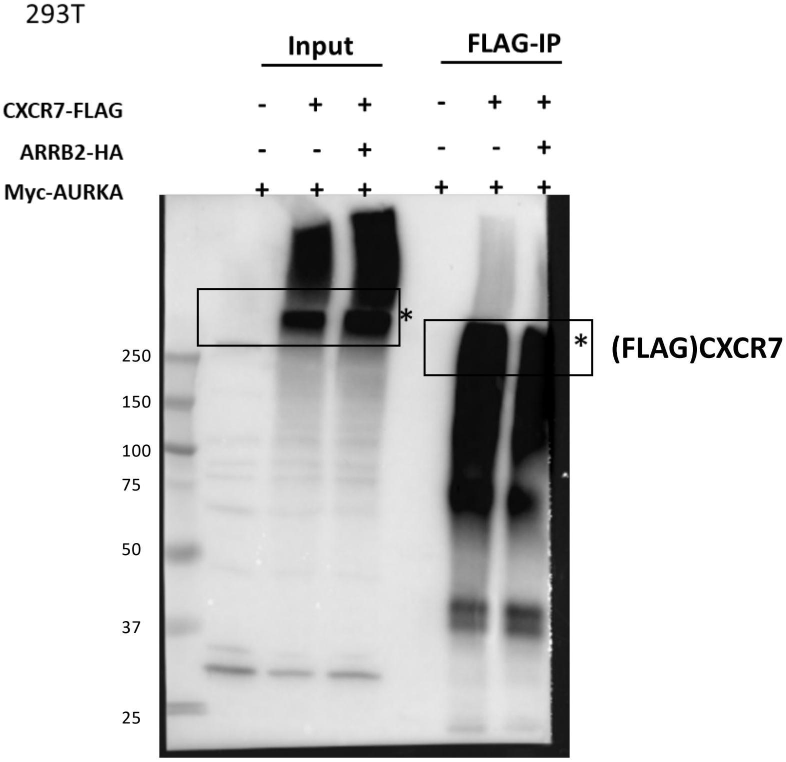

* CXCR7- FLAG becomes aggregated during WB samples preparation and becomes accumulates at the border between stacking/resolving gels

Uncropped images of immunoblots relative to Figure 4. Black boxes indicate where images were cropped.

C

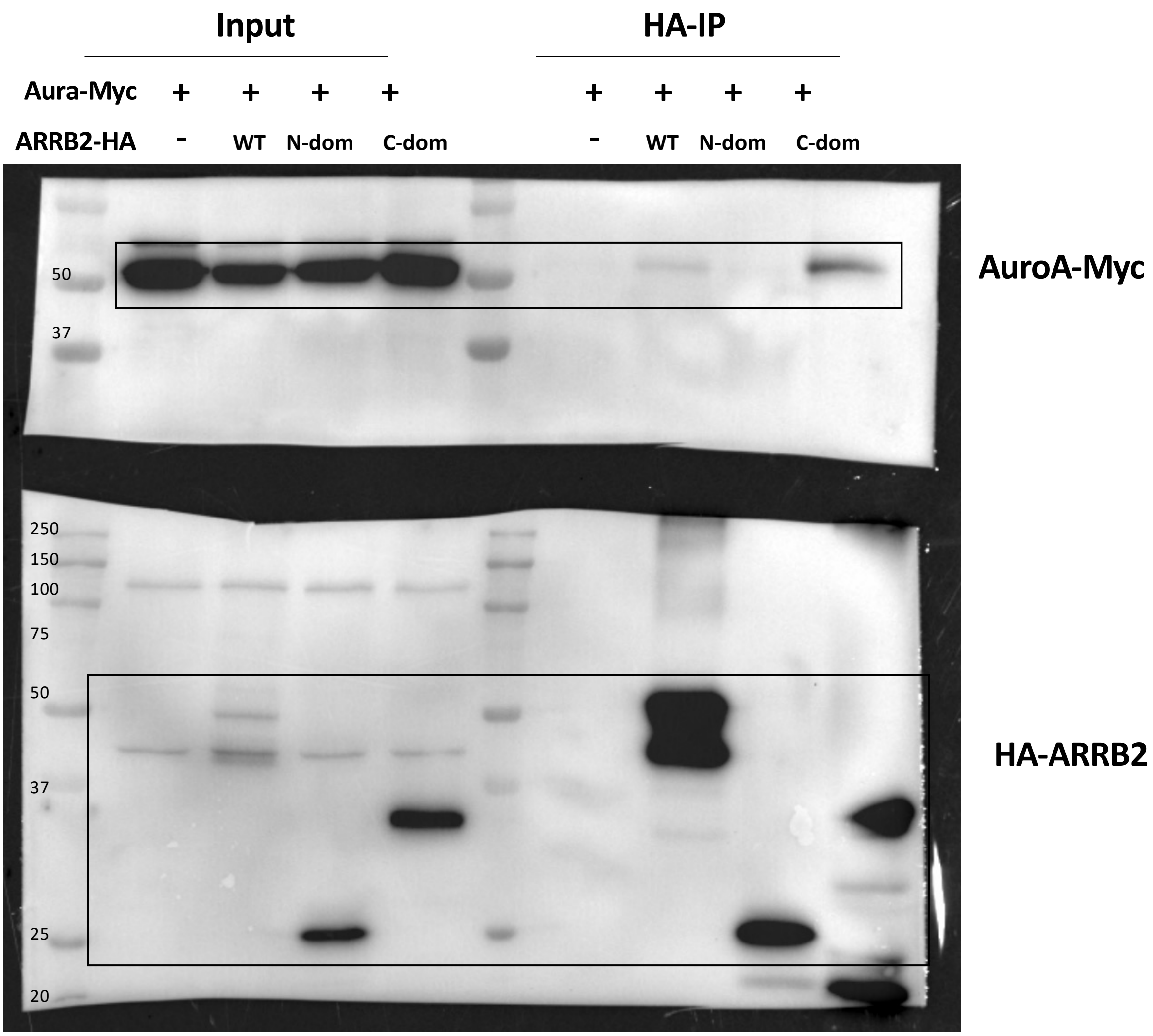

Uncropped images of immunoblots relative to Figure 4. Black boxes indicate where images were cropped.

D

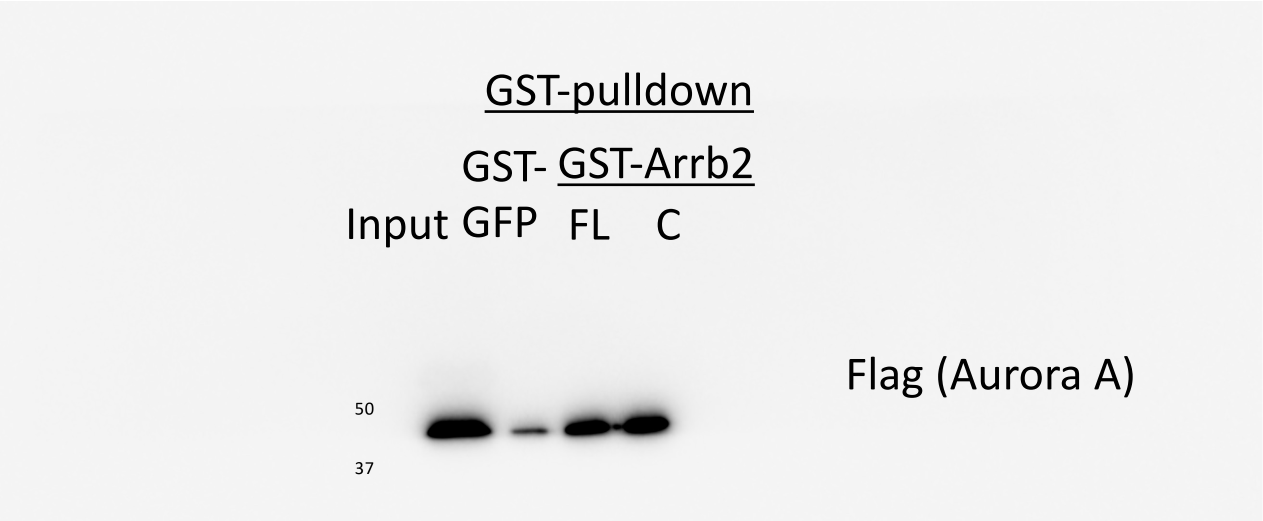

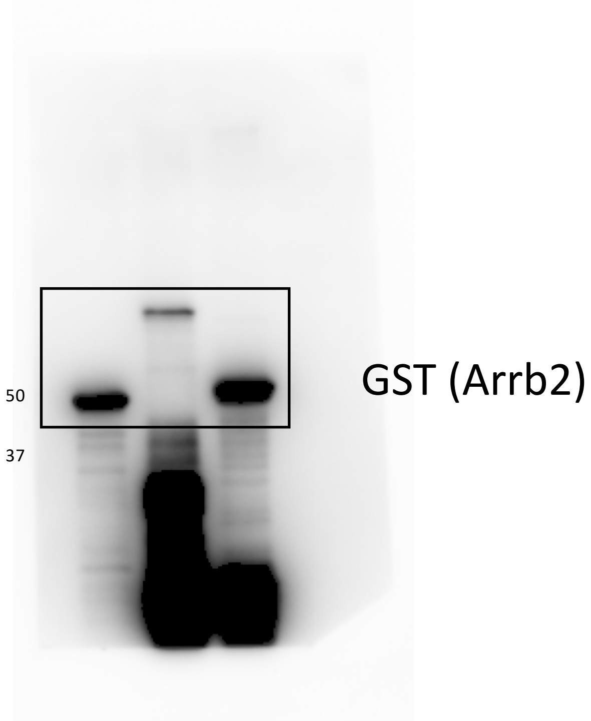

Uncropped images of immunoblots relative to Figure 4. Black boxes indicate where images were cropped.

E

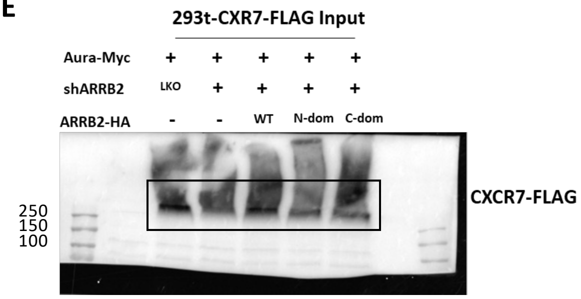

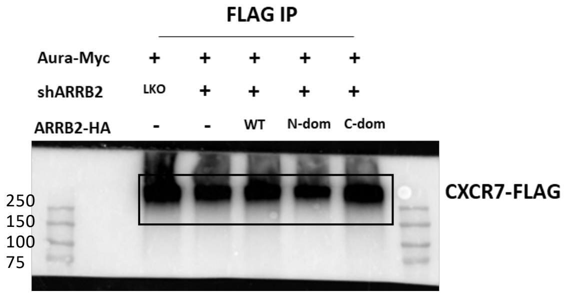

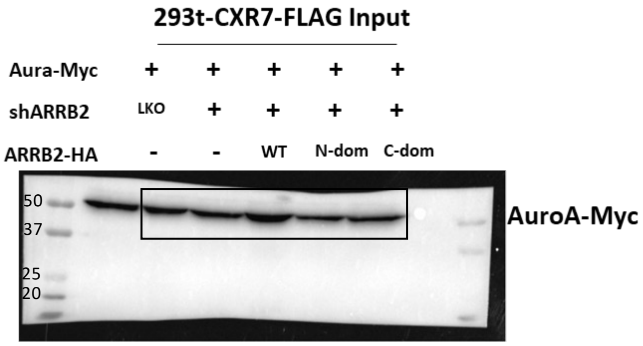

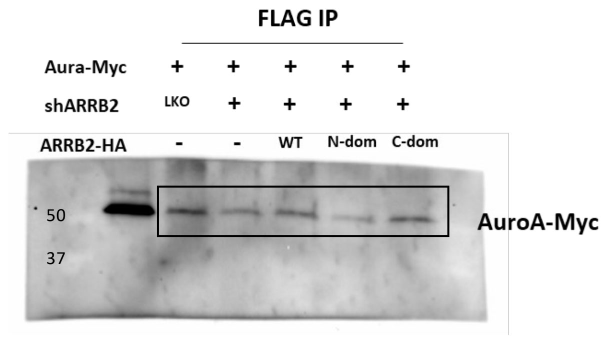

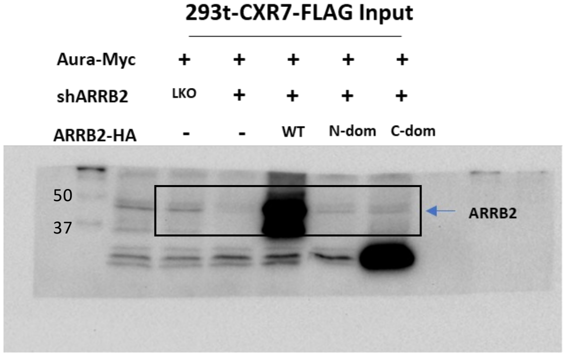

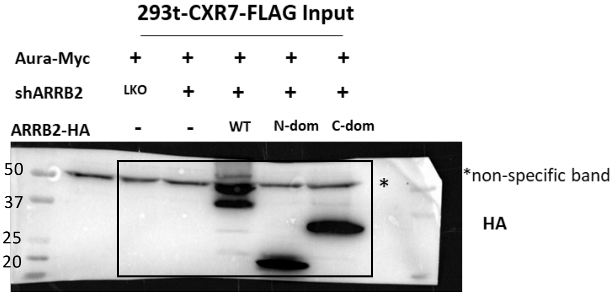

*CXCR7 becomes aggregated during IP preparation and accumulates at the border between stacking/resolving gels

Uncropped images of immunoblots relative to Figure 4. Black boxes indicate where images were cropped.

F

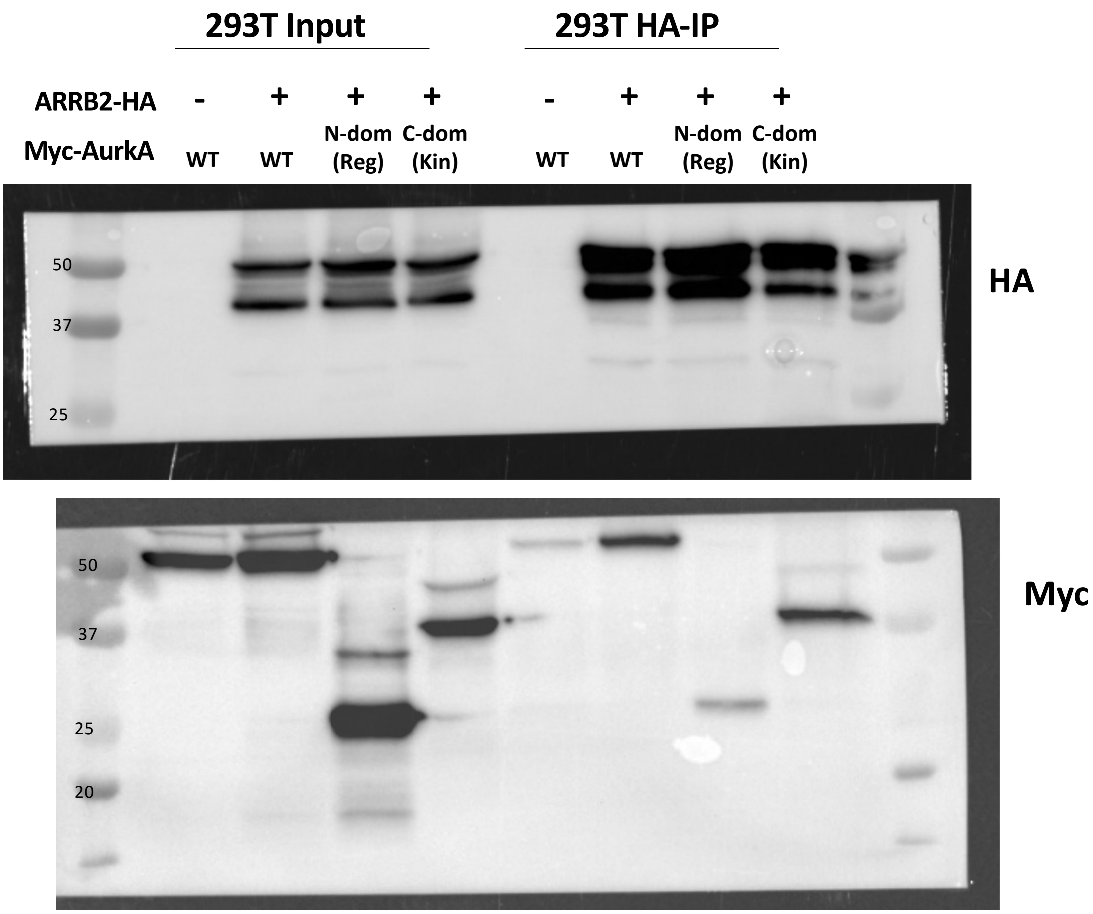

Uncropped images of immunoblots relative to Figure 4. Black boxes indicate where images were cropped.

G

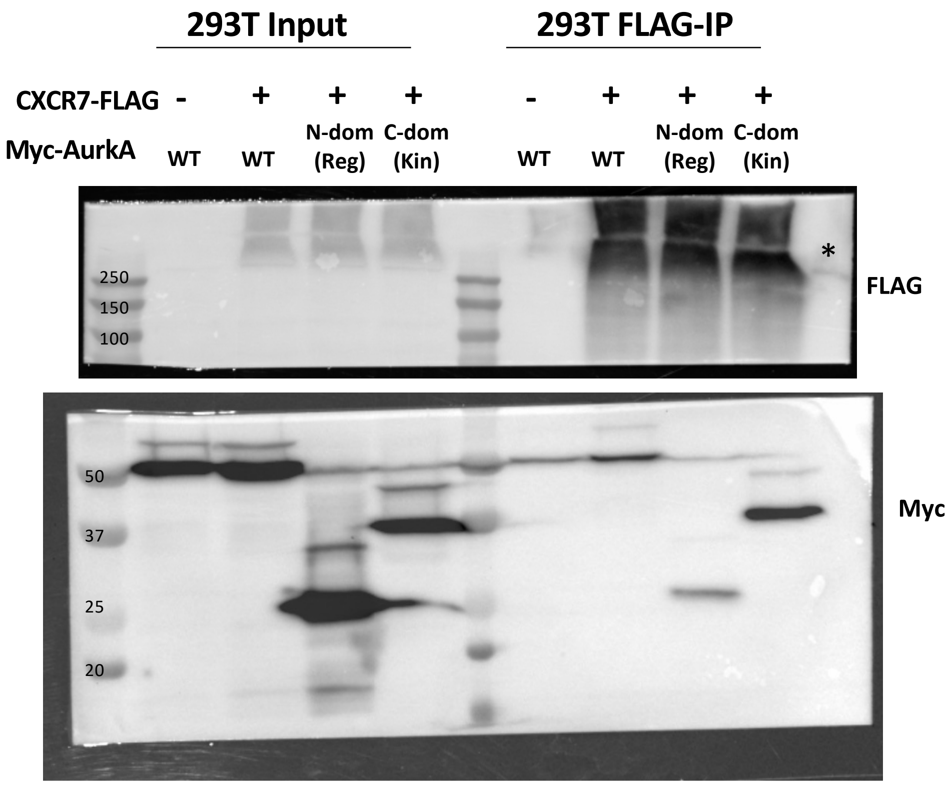

*CXCR7 becomes aggregated during IP preparation and accumulates at the border between stacking/resolving gels

Uncropped images of immunoblots relative to Figure 4. Black boxes indicate where images were cropped.

H

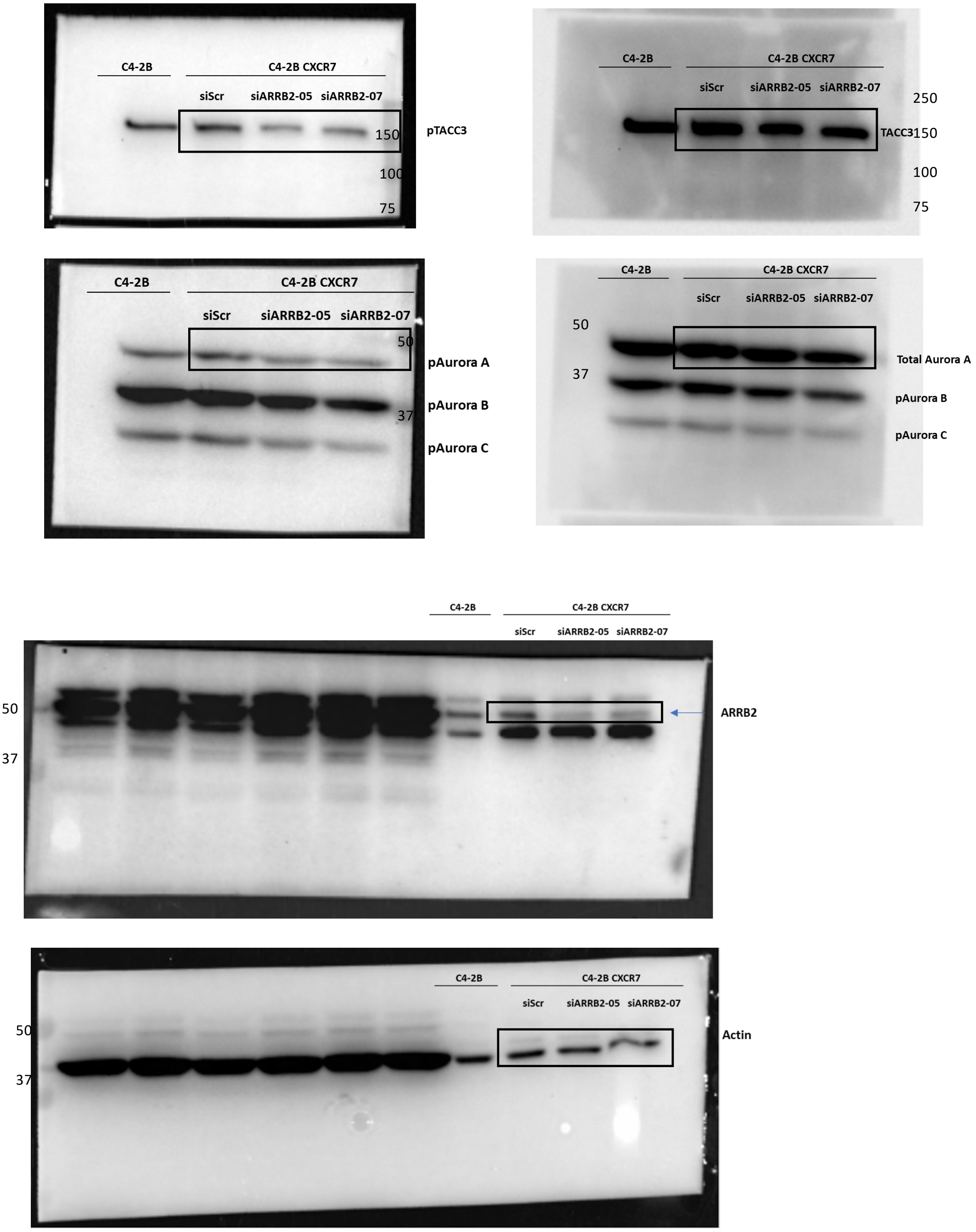

Uncropped images of immunoblots relative to Figure S4. Black boxes indicate where images were cropped.

A

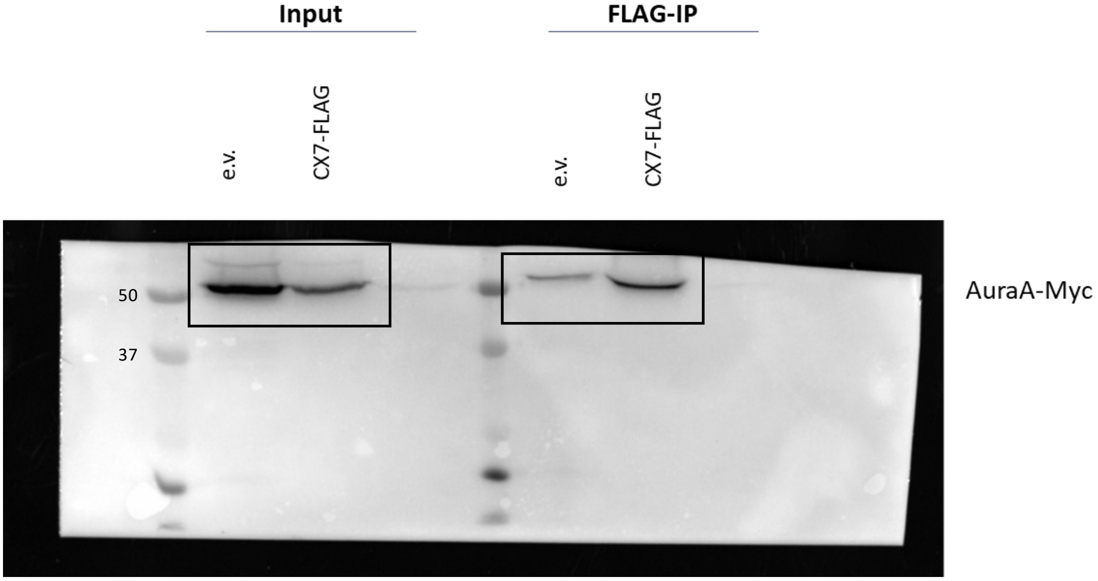

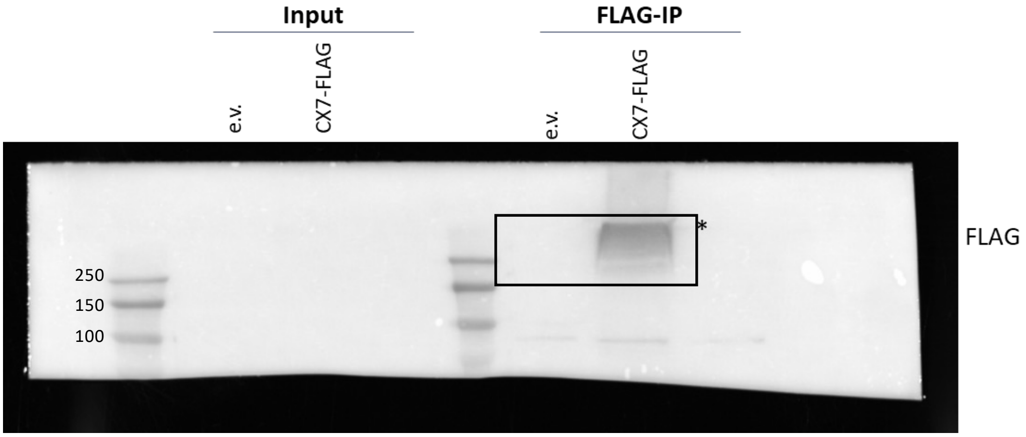

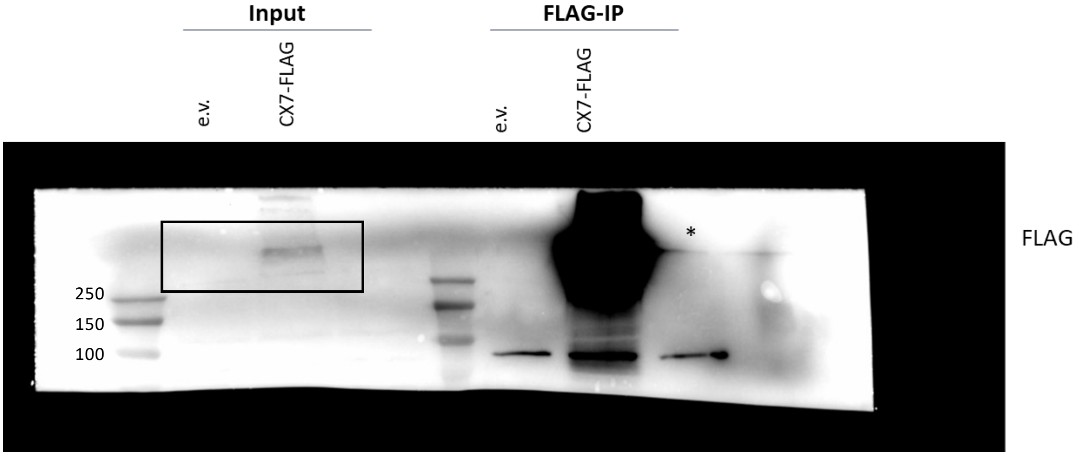

* CXCR7- FLAG becomes aggregated during WB samples preparation and becomes accumulates at the border between stacking/resolving gels

Uncropped images of immunoblots relative to Figure S4. Black boxes indicate where images were cropped.

B

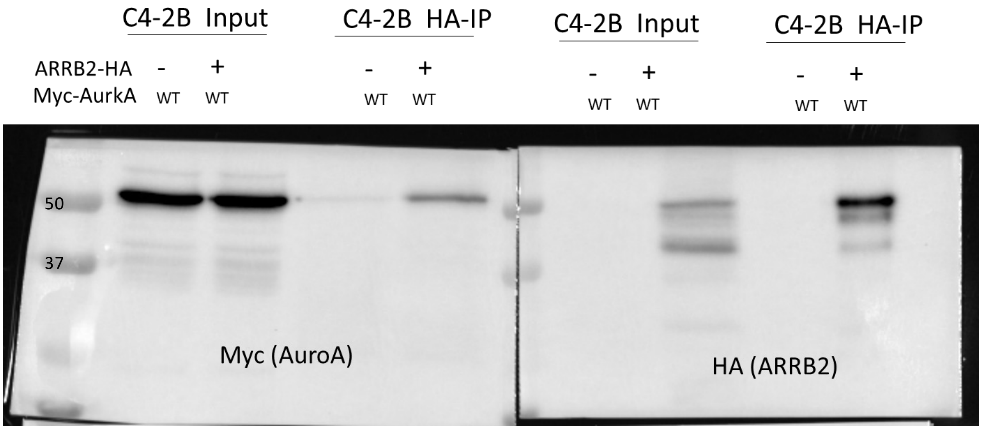

Uncropped images of immunoblots relative to Figure 5F. Black boxes indicate where images were cropped.

F

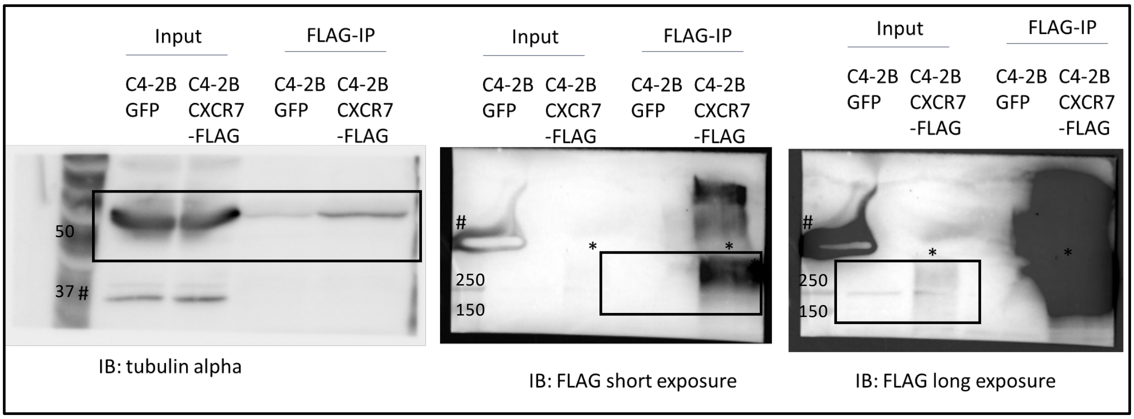

*CXCR7 becomes aggregated during IP preparation and accumulates at the border between stacking/resolving gels

#non-specific band

Uncropped images of immunoblots relative to Figure 6D. Black boxes indicate where images were cropped.

D

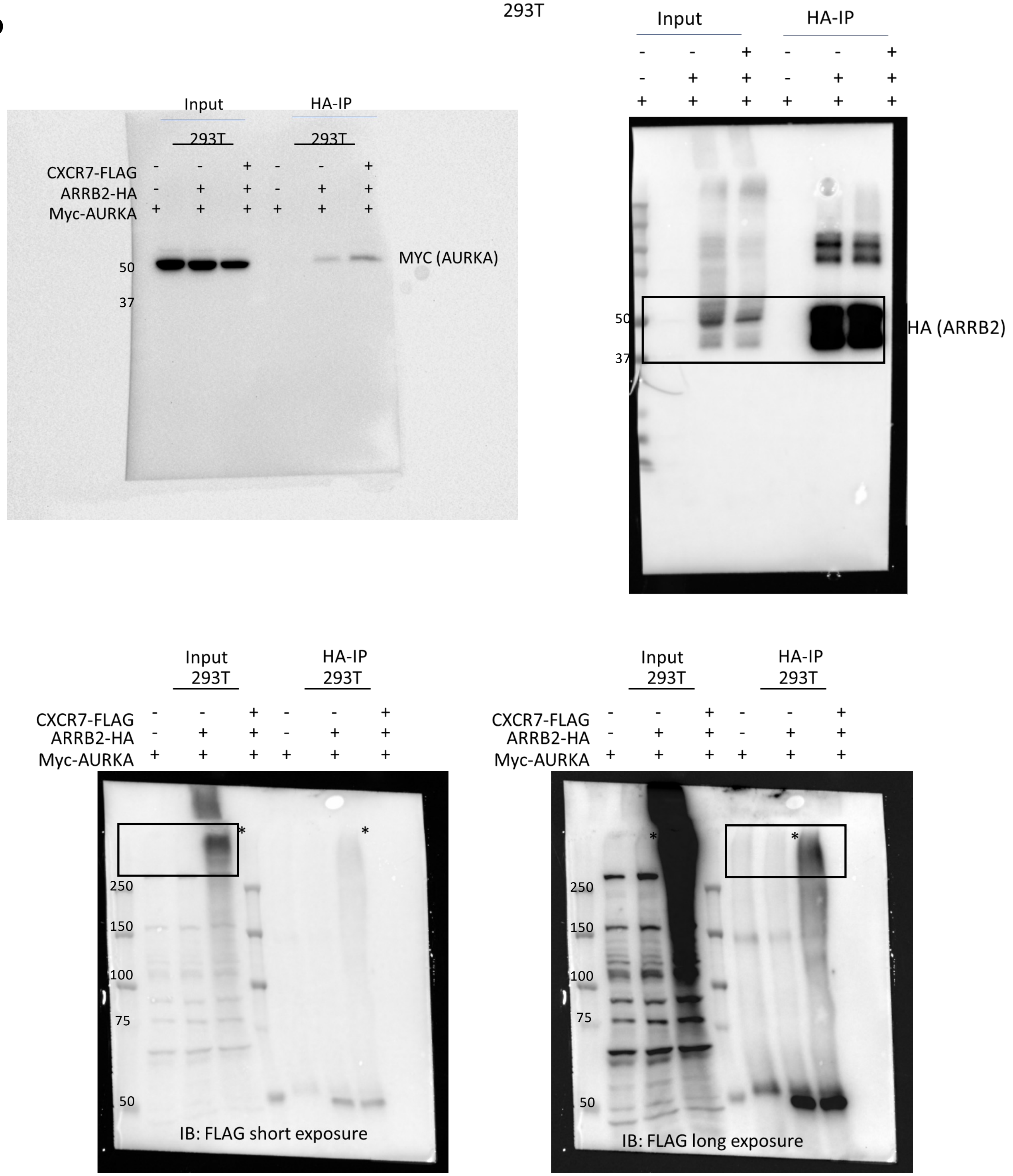

* CXCR7- FLAG becomes aggregated during WB samples preparation and accumulates at the border between stacking/resolving gels

Uncropped images of immunoblots relative to Figure 6E. Black boxes indicate where images were cropped.

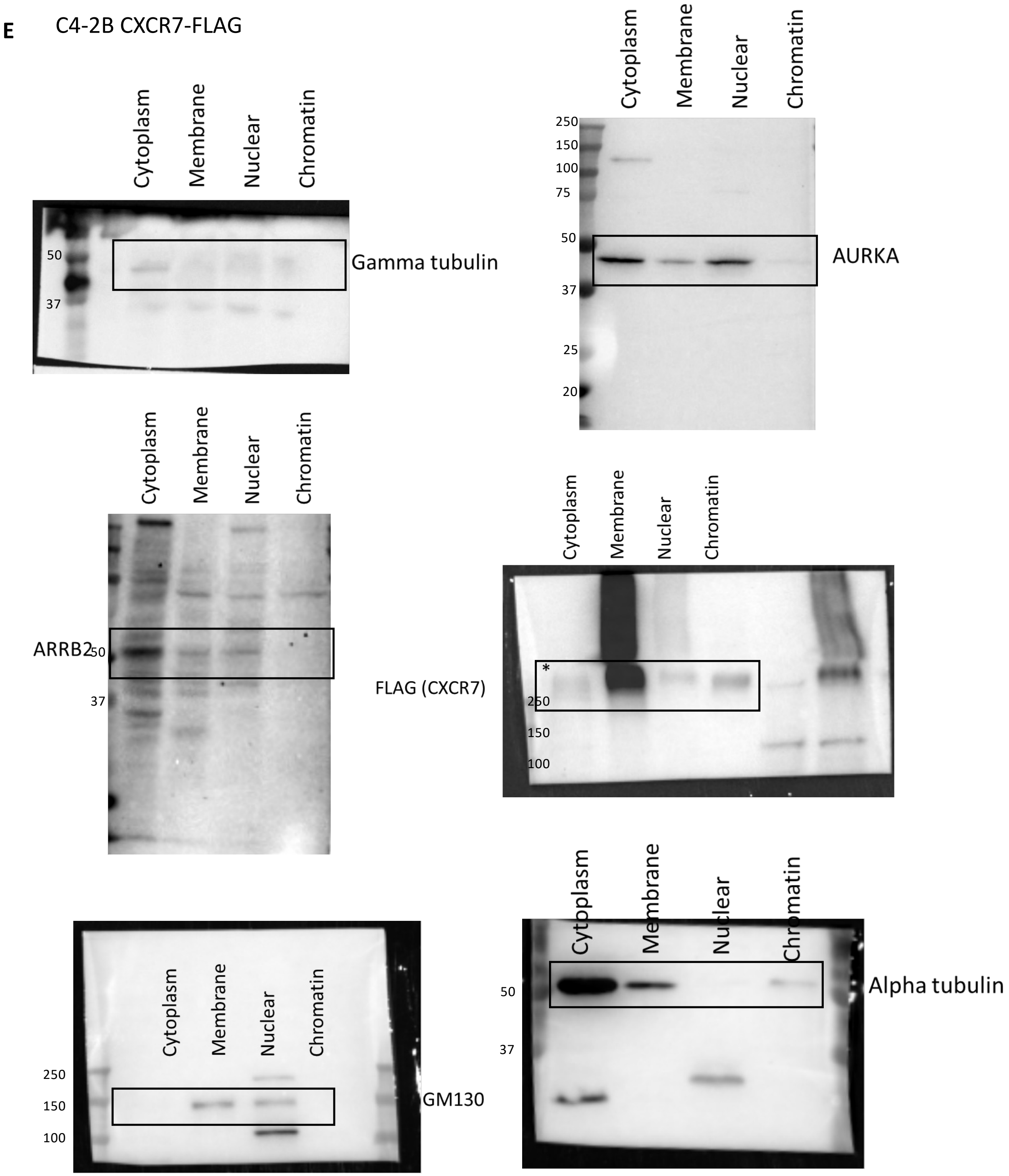

CXCR7- FLAG becomes aggregated during WB samples preparation and accumulates at the border between stacking/resolving gels

Uncropped images of immunoblots relative to Figure 6E. Black boxes indicate where images were cropped.

E

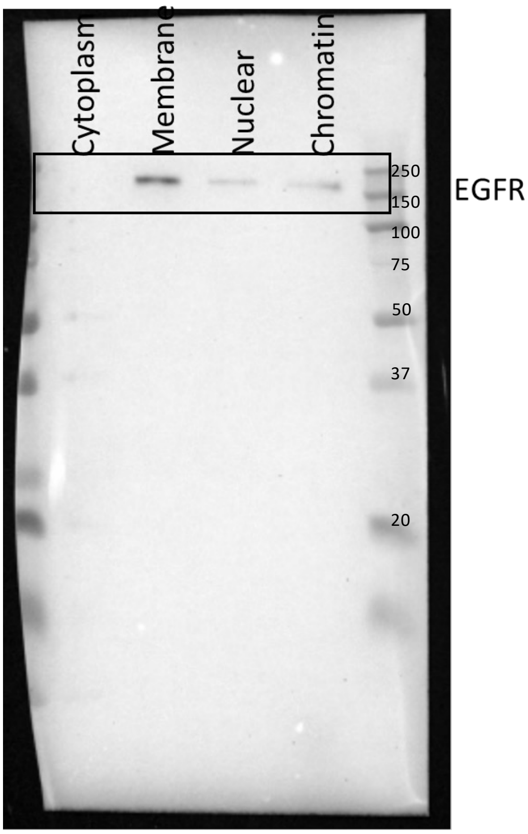

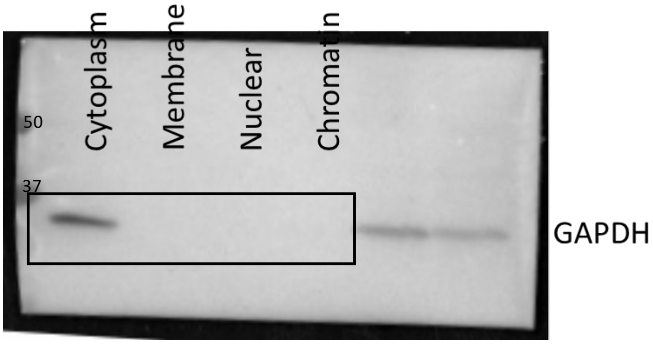

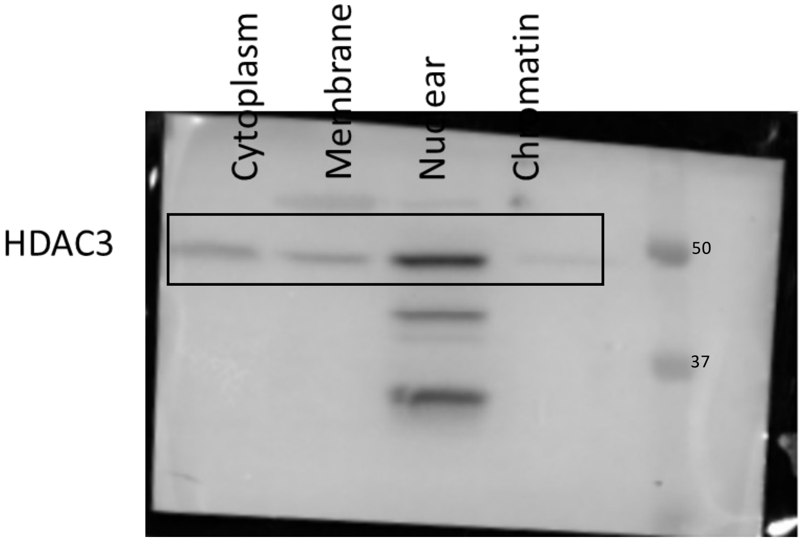

Uncropped images of immunoblots relative to Figure S6A. Black boxes indicate where images were cropped.

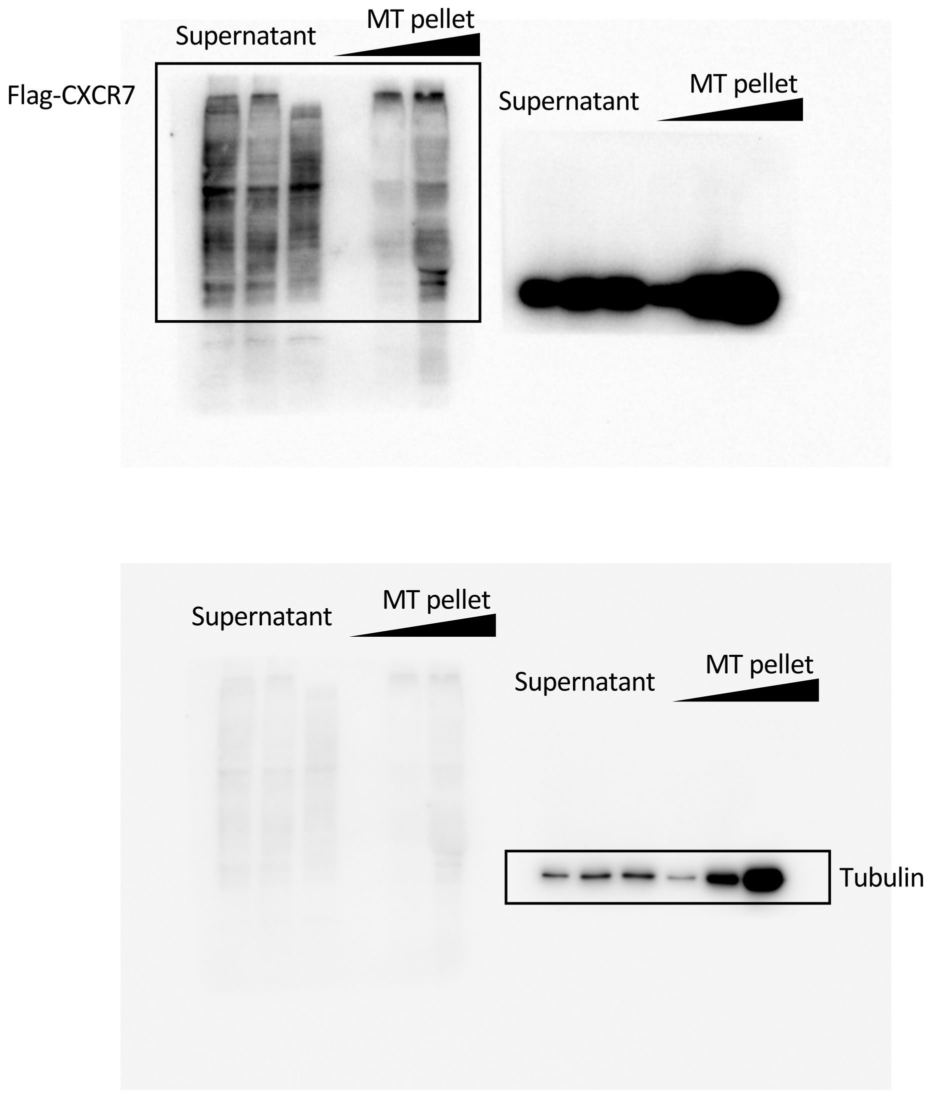

Supplement: Supplemental data [file jci-133-166248-s022.pdf]
